# Comparative Genomic Analysis of *Mycobacteriaceae* Reveals Horizontal Gene Transfer-Mediated Evolution of the CRISPR-Cas System in the Mycobacterium tuberculosis Complex

**DOI:** 10.1128/mSystems.00934-20

**Published:** 2021-01-19

**Authors:** Anoop Singh, Mohita Gaur, Vishal Sharma, Palak Khanna, Ankur Bothra, Asani Bhaduri, Anupam Kumar Mondal, Debasis Dash, Yogendra Singh, Richa Misra

**Affiliations:** a Department of Zoology, University of Delhi, Delhi, India; b CSIR-IGIB, Sukhdev Vihar, New Delhi, India; c Cluster Innovation Centre, University of Delhi, Delhi, India; d Department of Zoology, Sri Venkateswara College, University of Delhi, Delhi, India; University of Southampton

**Keywords:** CRISPR-Cas system, *Mycobacterium*, *Mycobacterium tuberculosis*, *Mycobacterium canettii*, horizontal gene transfer, IS*6110*, evolution, CRISPR-Cas system, comparative genomics, transposons

## Abstract

Clustered regularly interspaced short palindromic repeats (CRISPR) and CRISPR-associated (Cas) genes are conserved genetic elements in many prokaryotes, including Mycobacterium tuberculosis, the causative agent of tuberculosis. Although knowledge of CRISPR locus variability has been utilized in M. tuberculosis strain genotyping, its evolutionary path in *Mycobacteriaceae* is not well understood. In this study, we have performed a comparative analysis of 141 mycobacterial genomes and identified the exclusive presence of the CRISPR-Cas type III-A system in M. tuberculosis complex (MTBC). Our global phylogenetic analysis of CRISPR repeats and Cas10 proteins offers evidence of horizontal gene transfer (HGT) of the CRISPR-Cas module in the last common ancestor of MTBC and Mycobacterium canettii from a *Streptococcus*-like environmental bacterium. Additionally, our results show that the variation of CRISPR-Cas organization in M. tuberculosis lineages, especially in the Beijing sublineage of lineage 2, is due to the transposition of insertion sequence IS*6110*. The direct repeat (DR) region of the CRISPR-Cas locus acts as a hot spot for IS*6110* insertion. We show in M. tuberculosis H37Rv that the repeat at the 5′ end of CRISPR1 of the forward strand is an atypical repeat made up partly of IS-terminal inverted repeat and partly CRISPR DR. By tracing an undetectable spacer sequence in the DR region, the two CRISPR loci could theoretically be joined to reconstruct the ancestral single CRISPR-Cas locus organization, as seen in M. canettii. This study retracing the evolutionary events of HGT and IS*6110*-driven genomic deletions helps us to better understand the strain-specific variations in M. tuberculosis lineages.

**IMPORTANCE** Comparative genomic analysis of prokaryotes has led to a better understanding of the biology of several pathogenic microorganisms. One such clinically important pathogen is M. tuberculosis, the leading cause of bacterial infection worldwide. Recent evidence on the functionality of the CRISPR-Cas system in M. tuberculosis has brought back focus on these conserved genetic elements, present in many prokaryotes. Our study advances understanding of mycobacterial CRISPR-Cas origin and its diversity among the different species. We provide phylogenetic evidence of acquisition of CRISPR-Cas type III-A in the last common ancestor shared between MTBC and *M. canettii*, by HGT-mediated events. The most likely source of HGT was an environmental *Firmicutes* bacterium. Genomic mapping of the CRISPR loci showed the IS*6110* transposition-driven variations in M. tuberculosis strains. Thus, this study offers insights into events related to the evolution of CRISPR-Cas in M. tuberculosis lineages.

## Abstract (precis)

Comparative genomic analysis of prokaryotes has led to a better understanding of the biology of several pathogenic microorganisms. One such clinically important pathogen is M. tuberculosis, the leading cause of bacterial infection worldwide.

## INTRODUCTION

The discovery of CRISPR-Cas (clustered regularly interspaced short palindromic repeats–CRISPR-associated proteins) system in Mycobacterium tuberculosis complex (MTBC) has been an important clinical finding for epidemiological studies (1–3). The CRISPR-Cas genomic locus has been frequently employed for strain genotyping (spoligotyping) in M. tuberculosis, the most dreaded infectious organism of MTBC (4). M. tuberculosis is the causative agent of tuberculosis (TB) and infects more than one-quarter of the world’s population (5). MTBC was earlier grouped with environmental mycobacteria within a single genus, *Mycobacterium*. However, a new classification system proposed by Gupta et al. revisits the taxonomy and divides the mycobacterial species into five distinct clades based on the conserved signature indels and proteins (6). The *Mycobacterium* genus is now emended to encompass only the “Tuberculosis-Simiae” clade, which includes the group of slow-growing MTBC pathogens and nontuberculous mycobacteria (NTMs). MTBC comprises of human-adapted lineages (lineages 1 to 4 and lineage 7) of M. tuberculosis *sensu stricto*, M. tuberculosis variant africanum (lineages 5 and 6), and the recently discovered M. tuberculosis RW-TB008 (lineage 8), known for its early divergence from the rest of MTBC members (7). Besides these lineages, several animal-adapted forms, including M. tuberculosis variant bovis, M. tuberculosis variant caprae, M. tuberculosis variant microti, M. tuberculosis variant pinnipedii, M. tuberculosis variant origys, M. tuberculosis variant mungi, M. tuberculosis variant suricattae, M. tuberculosis variant dassie, and M. tuberculosis variant chimpanzee, are also included in MTBC (8). In addition to these classical members of MTBC, some studies occasionally include Mycobacterium canettii strains, also known as smooth tubercle bacilli (STBs), in MTBC based on nucleotide identity, but the present study includes only the classical members in “MTBC” (9). MTBC is said to evolve from an M. canettii-like ancestor that had an environmental reservoir (10). The other four novel genera are *Mycolicibacterium* gen. nov., *Mycolicibacter* gen. nov., *Mycolicibacillus* gen. nov., and *Mycobacteroides* gen. nov. corresponding to the “Fortuitum-Vaccae,” “Terrae,” “Triviale,” and “Abscessus-Chelonae” clades, respectively (6).

In recent years, the CRISPR-Cas system has garnered a lot of attention in other prokaryotes such as Streptococcus mutans, Pseudomonas aeruginosa, etc., with mounting evidence on its physiological roles like gene regulation, virulence, evolutionary adaptation apart from the classical role in evasion and defense against phage predation (11, 12). Additionally, the recent evidence on the activity of M. tuberculosis CRISPR interference system in invader defense and potential for an active genome editing system (13, 14) has brought the focus back to the MTBC CRISPR-Cas system. The most defining feature of the CRISPR-Cas locus is the presence of a CRISPR array, comprising of short direct repeats (DR) separated by short variable DNA sequence “spacers” and flanked by *cas* genes (15). CRISPR array with no adjacent *cas* genes is known as orphan CRISPR (11, 15). Based on the effector module composition, CRISPR-Cas systems are classified into two classes with six types (types I to VI) and 33 subtypes (16). Considerable diversity of CRISPR-Cas systems exists among various prokaryotic species, possibly owing to the selective environmental and/or host pressure (17). The organization of MTBC CRISPR-Cas type III-A system is considered mostly conserved with two CRISPR loci and the *cas* gene cluster of nine genes: *cas6*, *cas10* (*csm1*), *csm2*, *csm3*, *csm4*, *csm5*, *csm6*, *cas1*, and *cas2* adjacent to the CRISPR1 locus (2, 13). However, some clinical isolates, particularly belonging to M. tuberculosis lineage 2 strains (Beijing sublineage), show deletion in the CRISPR-Cas locus (18). The Beijing sublineage represents one of the most virulent and drug-resistant clusters among M. tuberculosis isolates. This lineage also possesses a remarkably high proportion of MTBC-specific insertion sequences (IS), IS*6110*, which are widely used as an epidemiological marker for TB (19). IS*6110* belongs to the IS*3* family of IS, comprising of a 1,361-bp sequence with 28-bp terminal inverted repeats (IR) and 3-bp DR of target sequences at its extremities. The IS*6110* sequence contains two partially overlapping open reading frames (ORFs), *orfA* and *orfB* encoding transposases (20). Expansion of IS is considered a key feature in the MTBC genome reduction process and is found at multiple sites in the genome, with one of the insertion sites located in the CRISPR-Cas locus (19). However, the impact of IS*6110* transposition on the evolution of the CRISPR-Cas system in M. tuberculosis has not been studied yet.

An earlier study reported similarities in the MTBC CRISPR-Cas type III-A system with some *M. canettii* strains but not with any NTMs, suggesting a horizontal gene transfer (HGT)-related acquisition (21); however, little is known about this evolutionary adaptation. Here, we performed a comprehensive comparative genomic analysis of 141 mycobacterial genomes, available at NCBI-RefSeq, to advance our understanding of the origin of the mycobacterial CRISPR-Cas system, its diversity, and interrelation among species with reference to the recent reclassification of *Mycobacteriaceae* genomes (6). Our results offer strong phylogenetic evidence of a HGT-mediated acquisition of CRISPR-Cas type III-A system in MTBC from an environmental *Firmicutes* as the likely source. Additionally, the analysis shows the influence of IS*6110* transposition on the M. tuberculosis CRISPR-Cas system. Therefore, a deeper look into this genomic region gave fresh insights on the evolution of the CRISPR-Cas system in MTBC, especially in M. tuberculosis strains.

## RESULTS AND DISCUSSION

### Diversity of CRISPR-Cas systems in *Mycobacteriaceae* and potential targets of CRISPR spacers.

To characterize CRISPR-Cas systems in *Mycobacteriaceae*, 141 genome sequences from NCBI-RefSeq were analyzed in the present study (see Table S1a in the supplemental material). The presence of true CRISPRs in the genome was assessed by the CRISPRCasFinder tool using default parameters (22). To discriminate spurious CRISPR-like elements from the true CRISPRs, only CRISPRs classified with evidence levels 3 and 4 were considered for further analyses. Based on the selection criteria, in total, 36 CRISPR loci/arrays containing 891 spacers in 19 genomes were selected. Among these predicted arrays, five CRISPR arrays are of evidence level 3, whereas the other 31 CRISPR arrays were assigned to level 4, projecting them as high-confidence CRISPR candidates (Table S1b).

10.1128/mSystems.00934-20.1 TABLE S1 141 mycobacterial genomes and predicted CRISPR locus. (a) Detailed list of 141 mycobacterial genomes. (b) CRISPRCasFinder result of mycobacterial genomes. Download Table S1, XLSX file, 0.04 MB. Copyright © 2021 Singh et al. 2021 Singh et al. This content is distributed under the terms of the Creative Commons Attribution 4.0 International license.

**FIG 1:**
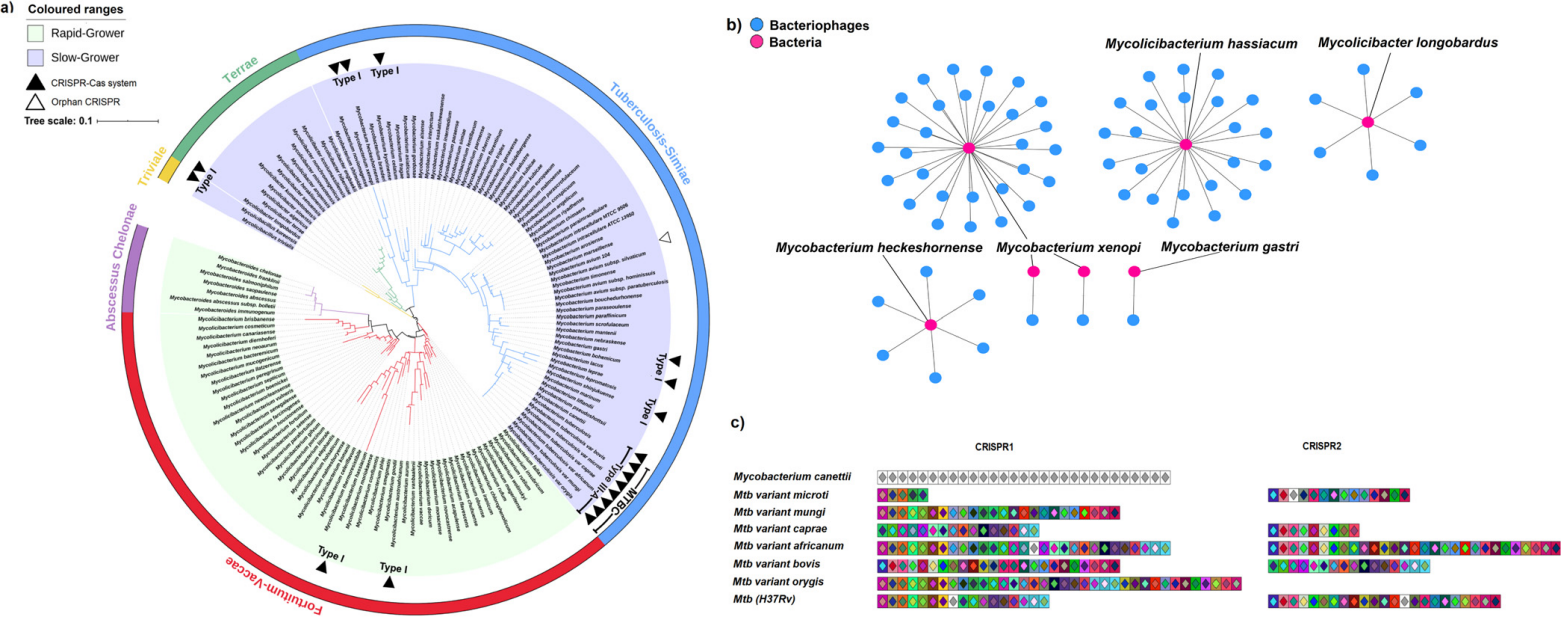
Analysis of CRISPR-Cas systems in *Mycobacteriaceae* reveals exclusive presence of type III-A system in MTBC and the interrelatedness of MTBC CRISPR spacers. (a) 16S rRNA gene-based phylogeny of *Mycobacteriaceae* shows the differential distribution of CRISPR-Cas systems. The different monophyletic clades/genera are represented by a color strip and colored branches. The presence of true CRISPR-Cas systems is illustrated as filled triangles, while the hollow triangle depicts an orphan CRISPR. The classification types are mentioned along with the triangles. The inner circle is color coded as light green and light purple, representing rapid-growing and slow-growing bacteria, respectively. (b) Mycobacteriophages as potential targets of CRISPR spacers. The phage-bacterium bipartite network derived from *Mycobacteriaceae* spacer sequences and their matches in the phage genome sequences showing mycobacteriophages targeted by the spacers of Mycolicibacterium hassiacum, Mycolicibacter longobardus, Mycobacterium heckeshornense, Mycobacterium xenopi, and Mycobacterium gastri. Pink nodes, bacteria; blue nodes, bacteriophages; edges, shared spacer-protospacer pair. (c) Comparative genomic analysis of MTBC and *M. canettii* CRISPR spacer content. The complete CRISPR loci are illustrated as two clusters of CRISPR1 and CRISPR2. Each color-coded box differentiates groups of spacer sequences. The unique spacers are depicted as gray boxes, and similar spacers are marked with the same colors across the data set. *Mtb*, Mycobacterium tuberculosis.

Further, to correctly determine the presence of CRISPR-Cas systems in all 141 genomes, Cas proteins were identified using a combination of CRISPRCasFinder and HMMER 3 search against a collection of 395 Cas protein profiles obtained from a previous study (15). The results revealed the presence of true CRISPR-Cas system in 18 genomes (12 species) and an orphan CRISPR locus in one genome (Mycobacterium avium) (Fig. 1a). The distribution and diversity of the CRISPR-Cas systems in *Mycobacteriaceae* is shown along a phylogenetic tree generated using 16S rRNA sequences from the sequenced genomes (Fig. 1a). Our analysis revealed the presence of five monophyletic clades, in accordance with the new classification system by Gupta et al. (6) (Fig. 1a and Table S1a). We observed that CRISPR-Cas loci are predominantly present in the slow-growing monophyletic clade, Tuberculosis-Simiae. Among the 12 species that possess a true CRISPR-Cas system, eight species belong to the *Mycobacterium* genus. On the basis of the presence of signature *cas* genes, *cas3* and *cas10*, the CRISPR-Cas system was further classified, and out of the eight species of the *Mycobacterium* genus, six species were found to possess the type I system and the remaining two species, belonging to MTBC (seven members) and *M. canettii*, exclusively possess the type III-A system (Fig. 1a and Table S1a). The presence and organization of MTBC type III-A CRISPR-Cas system are as described in earlier reports (2, 13); however, the present study expands the search to 141 mycobacterial genomes, including 129 species compared to the 22 genomes, including 14 species from the earlier study (2). Among the other four genera, two species of *Mycolicibacterium* and one species each in *Mycolicibacter* and *Mycolicibacillus*, respectively, show the presence of a type I system. However, genomes of *Mycobacteroides* lack any CRISPR-Cas system (Fig. 1a). The detailed features of the CRISPR DR were predicted using the CRISPRmap program (23). The features included consensus sequences, secondary structures, conserved motifs, family, and superclass. These features are generally specific to a particular type/subtype of CRISPR-Cas system irrespective of the bacterial/archaeal species harboring them. Comparison of these conserved features among the 141 genomes revealed that all MTBC members and *M. canettii* (STB-A) share a conserved consensus CRISPR DR belonging to the same family and superclass (Table S2).

10.1128/mSystems.00934-20.2 TABLE S2 CRISPRmap results showing motifs, family, and superclass of CRISPR repeats of mycobacterial genomes. Download Table S2, DOCX file, 0.3 MB. Copyright © 2021 Singh et al. 2021 Singh et al. This content is distributed under the terms of the Creative Commons Attribution 4.0 International license.

To look for potential targets of *Mycobacteriaceae* CRISPR spacers, we performed a command-line NCBI-BLASTN with 90% identity and 90% query coverage against the NCBI phage and plasmid databases, with the spacer sequences extracted from CRISPR arrays. Several putative protospacers homologous with the spacers in the phage and plasmid genomes were identified (Table S3a and S3b). We identified the targets in a few known mycobacteriophages such as Bxz1, Anaya, Iracema64, L5, etc., for spacer sequences of Mycolicibacterium hassiacum, Mycolicibacter longobardus, Mycobacterium heckeshornense, Mycobacterium xenopi, and Mycobacterium gastri (Fig. 1b and Table S3a). Although MTBC and *M. canettii* together possess ∼53% of CRISPR arrays in the *Mycobacterium* genus, we did not find any significant similarity with any phage or plasmid database sequence. This could be due to the limited sequencing data available for uncharacterized mycobacteriophages. To overcome this issue and understand the possible origin of spacer sequences in MTBC, we looked for the conservation pattern of spacer sequences in MTBC. Most spacer sequences were conserved within or across CRISPR arrays in the MTBC members with a low proportion of unique spacers. However, we observed that *M. canettii* spacer sequences were unique and did not match with any of the MTBC members (Fig. 1c). CRISPR spacers are acquired in response to exposure to foreign invading genetic elements, which results in sequence-specific memory, protecting bacteria from future invasion (15). Therefore, lack of any shared spacer sequences may be due to the phylogenetic distance of *M. canettii* from MTBC members, which are mainly variants of M. tuberculosis species and so phylogenetically more related to each other (7, 21). Another possibility could be the inability of M. tuberculosis to incorporate new spacers, unlike *M. canettii* (2, 24).

10.1128/mSystems.00934-20.3 TABLE S3 BLASTN results of mycobacterial spacer sequence against NCBI phage (a) and plasmid (b) databases. Download Table S3, XLSX file, 0.02 MB. Copyright © 2021 Singh et al. 2021 Singh et al. This content is distributed under the terms of the Creative Commons Attribution 4.0 International license.

### Evidence of horizontal gene transfer of CRISPR-Cas type III-A system in MTBC.

**FIG 2:**
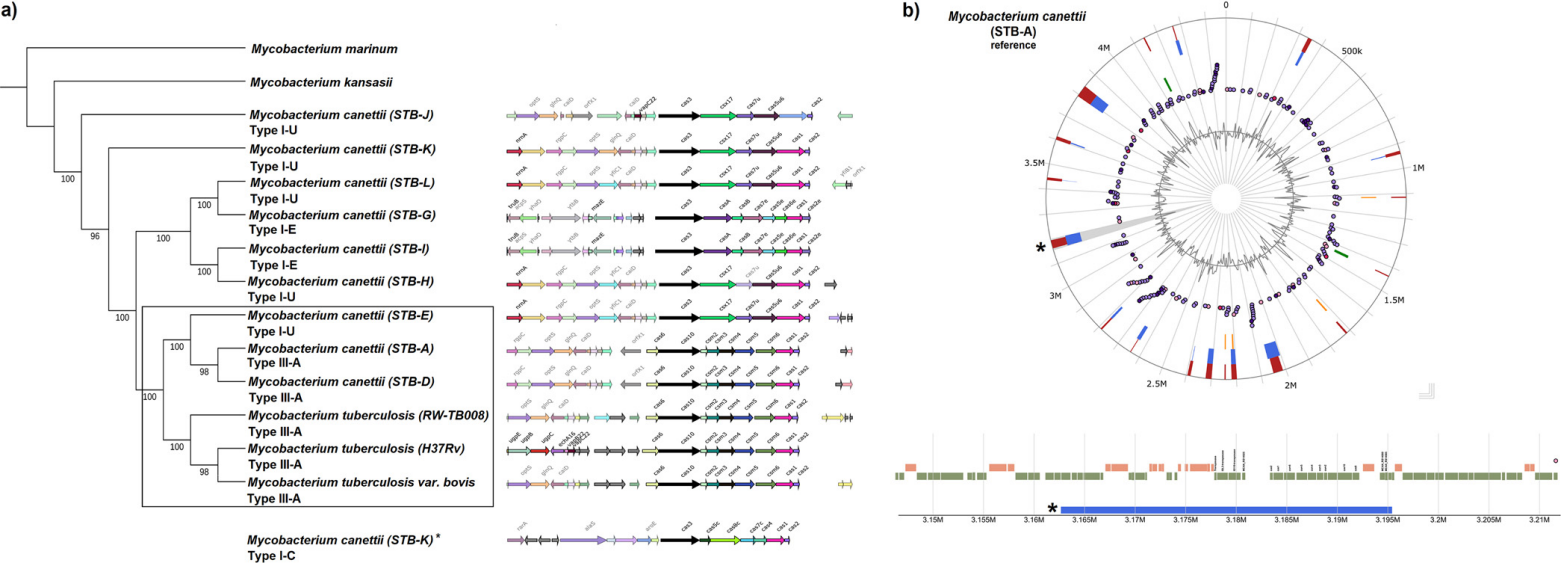
Phylogenetic relatedness of *M. canettii* strains with M. tuberculosis and genomic island visualization as evidence of horizontal transfer of CRISPR-Cas type III-A system in *M. canettii.* (a) Phylogenetic analysis reveals the presence of CRISPR type III-A system in M. tuberculosis and related STB strains. The core genome-based phylogenetic analysis included 9 STB strains, M. tuberculosis H37Rv and RW-TB008 strains, M. tuberculosis variant bovis, two closely related NTMs, M. kansasii and M. marinum (outgroup). The maximum likelihood method was employed to construct the tree based on core genome alignment. The bootstrap values are calculated using 1,000 replicates. The tree is supplemented on the right with the corresponding schematic representation of the CRISPR-Cas loci present in the corresponding mycobacterial genome. (b) A genomic island of the *M. canettii* genome shows the presence of the CRISPR-Cas locus. Circular and horizontal visualization of predicted genomic islands (GIs) in the *M. canettii* genome by IslandViewer 4 are shown. The distinct GIs are represented with colored blocks. Green, orange, blue, and red blocks correspond to the results obtained from IslandPick, SIGI-HMM, IslandPath-DIMOB, and the integrated results of the four tools, respectively. The virulence genes (purple for curated, light purple for homologs), antimicrobial resistance genes (pink for curated, light pink for homologs), and pathogenicity-related genes (orange) are shown as circular glyphs. The innermost ring shows the GC content. The GI of interest is highlighted in gray and marked by an asterisk, and the magnified horizontal view is shown at the bottom. The blue rectangular block marks the length of the genomic island. Green and orange blocks mark the gene annotation.

Past findings suggest that MTBC members evolved into obligate pathogens by a bimodal evolutionary process of reductive evolution and selective genome expansion as evident by comparative genome analyses of MTBC, STBs, and NTMs (25). Genomes of *M. canettii* strains (4.48 ± 0.05 Mb) are considered transitional forms between NTMs (6.4 to 6.6 Mb) and MTBC (4.4 Mb) (10), and unlike MTBC that has a clonal population (26), *M. canettii* strains are heterogeneous and have been described to frequently undergo HGT (21). Most of the gene acquisition related to patho-adaptation, virulence, and persistence in MTBC is suggested to occur via HGT in the last common ancestor of MTBC and *M. canettii* (27, 28). The same hypothesis is suggested for CRISPR-Cas system acquisition in MTBC (21). To examine this hypothesis and to understand the evolutionary path of the CRISPR-Cas system in MTBC, we generated a maximum likelihood phylogenetic tree based on core genome alignment of the reference genome of M. tuberculosis H37Rv, with nine STB strains (A, D, E, G, H, I, J, K, and L), an animal-adapted strain of M. tuberculosis variant bovis, M. tuberculosis RW-TB008 (lineage 8), and two most closely related NTMs, Mycobacterium kansasii and Mycobacterium marinum. M. tuberculosis RW-TB008 (lineage 8) was included in this phylogenetic tree analysis due to its intermediary phylogenetic position between *M. canettii* and other MTBC members, and the tree was rooted using M. marinum. STB strains are listed and described in Table S4a. The comparative genomics and phylogenetic analysis revealed a close relationship of M. tuberculosis and its variants with three STB strains (STB-A, STB-D, and STB-E) as they belonged to a single monophyletic clade (Fig. 2a). All the clade members possess a type III-A CRISPR-Cas system except STB-E that has type I-U (uncharacterized). The type I-U system was also found in the more distant STB-H, STB-J, STB-K, and STB-L strains (Fig. 2a). As reported earlier (21), an additional type I-C locus was found in STB-K, while STB-G and STB-I possessed type I-E system (Fig. 2a). This suggests that an independent evolutionary event may have resulted in the acquisition of type III-A CRISPR-Cas system in the last common ancestor of STB-A/D and MTBC. In view of the complete absence of CRISPR-Cas system in NTMs and the divergence seen in STB strains, independent HGT events seem to be the most plausible explanation for acquisition of CRISPR-Cas systems in various STB strains.

10.1128/mSystems.00934-20.4 TABLE S4 Data sets used in the analysis for HGT of the CRISPR-Cas type III-A system. (a) List of *M. canettii* strain genomes. (b) Clustering tree organism acronyms. (c) GenInfo identifiers (GI) of Cas10. Download Table S4, XLSX file, 0.03 MB. Copyright © 2021 Singh et al. 2021 Singh et al. This content is distributed under the terms of the Creative Commons Attribution 4.0 International license.

Traditionally, a horizontally transferred cluster of genes form syntenic blocks and observed as genomic islands (GIs) in recipient bacterial genomes (29). The GIs have been considered direct evidence of the HGT of genes playing a crucial role in the evolution of bacterial genomes (30). To predict the occurrence of GIs in STB-A (*M. canettii* reference genome), we used IslandViewer 4, which integrates four methods, SIGI-HMM, IslandPath-DIMOB, IslandPick, and Islander, to most accurately analyze the GIs in the genome (31). Out of the 19 GIs predicted by IslandViewer 4, one of the GIs in STB-A was found to possess CRISPR-Cas type III-A system, supporting the hypothesis of HGT-based CRISPR-Cas acquisition. The predicted GI is around 32,729 bp in length, possessing CRISPR-Cas type III-A system along with mobility genes such as transposase and integrase, as shown in Fig. 2b. A comparative GI prediction in M. tuberculosis genome revealed a smaller sized (16,446-bp) GI, a probable result of genomic reduction, carrying a *cas* gene and other mobility genes (see Fig. S1 in the supplemental material).

10.1128/mSystems.00934-20.6 FIG S1 Comparison of genomic island harboring the CRISPR-Cas system and other mobility genes between M. tuberculosis and *M. canettii.* IslandViewer 4 result shows prediction of genomic island (GI) in the M. tuberculosis genome (GenBank accession number NC_018143.2). GI predictions are represented as color blocks (integrated results in red, IslandPick in green, SIGI-HMM in orange, and IslandPath-DIMOB in blue). Virulence genes (purple for curated, light purple for homologs), antimicrobial resistance genes (pink for curated, light pink for homologs) and pathogenicity-related genes (orange) are shown as circular glyphs. The innermost ring shows the GC content. A GI of interest is highlighted in gray marked by an asterisk. The magnified horizontal view of the GI of interest marked with an asterisk is shown below the circular graph. Blue block marks the length of GI. Green and orange blocks mark the gene annotation. The comparative horizontal graph view of similar GIs in *M. canettii* is shown at the bottom. Download FIG S1, TIF file, 2.1 MB. Copyright © 2021 Singh et al. 2021 Singh et al. This content is distributed under the terms of the Creative Commons Attribution 4.0 International license.

**FIG 3:**
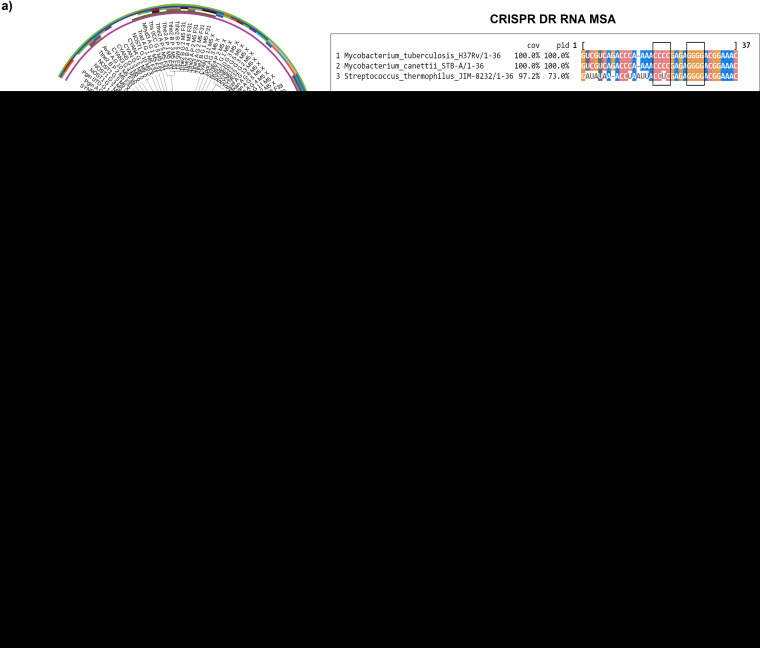
Phylogeny analysis based on CRISPR repeats and Cas10 predicts the acquisition source of the CRISPR-Cas system in the MTBC ancestor. (a) MTBC CRISPR repeat clusters with S. thermophilus cluster based on sequence and structural similarities. A hierarchical cluster tree was generated based on the multiple sequence-structure alignment of repeat sequences. The tree revealed a cluster of MTBC and *M. canettii* (highlighted in blue) along with S. thermophilus (highlighted in pale yellow). The right top panel shows the multiple sequence alignment of CRISPR DR RNA sequence of M. tuberculosis, *M. canettii*, and S. thermophilus. The conserved compatible bases, involved in RNA stem-loop formation, are shown inside rectangular boxes. The consensus MFE structure and sequence logo of aligned members from the cluster tree are shown in the right middle and right bottom panels, respectively. The conserved compatible bases involved in RNA stem formation are highlighted in similar color in the MFE structure. (b) Cas10 phylogeny shows evolutionary relatedness of MTBC with *Streptococcus* spp. The circular phylogenetic tree was generated from the global collection of Cas10 data obtained from the study of Makarova et al. (15). The bootstrap values are calculated from 1,000 replicates and are represented along the branches. The color strip represents different phyla. A magnified view of the area of interest is shown to the left of the circular tree highlighting the clustering of MTBC and *M. canettii* with *Streptococcus* species clade and Lactobacillus ruminis sharing a common ancestral node between them, indicating an HGT event.

To trace the origin of the STB-A GI, we used nucleotide BLAST but could not find a genus harboring such genomic loci. Therefore, to gain insight into the source of HGT, we analyzed the CRISPR repeats of MTBC and *M. canettii* using the CRISPRmap program. This program examines CRISPR repeat queries against the CRISPR repeat database to generate a clustering tree to determine the evolutionary relationships based on the conservation of CRISPR repeat sequence and similarities in their minimum free energy (MFE) secondary structures. The CRISPR repeat is a central regulatory element as it serves as the binding template for Cas proteins, and conservation of CRISPR DR RNA stem-loop structure is essential for interaction with effector complex, required for CRISPR biological function (23), as also recently shown in M. tuberculosis (13). Since MTBC members and *M. canettii* showed 100% conservation in their CRISPR DR, the consensus repeat was used as a query sequence in the program. Our results revealed clustering of the MTBC CRISPR cluster with Streptococcus thermophilus cluster (Fig. 3a, left panel, and Table S4b), suggesting a possible genetic exchange of CRISPR-Cas type III-A system between *Actinobacteria* and *Firmicutes*. Conservation in secondary structures of CRISPR RNA has been observed in diverse organisms that reflect conserved binding motifs and shared mechanisms of action of effector complex (23, 32). The consensus MFE structure of clustering tree members based on multiple sequence-structure alignment using LocARNA is shown in Fig. 3a, right middle panel, and a sequence logo of these aligned DR sequences, is shown in Fig. 3a, right bottom panel, respectively. The stem of the hairpin MFE structure shows the conserved compatible bases (highlighted in shades of green, blue, and yellow in Fig. 3a, right middle panel, and Fig. S2). The complete sequence-structure alignment file of these DR sequences from the cluster tree is shown in Fig. S2. Figure 3a, right top panel, shows an independent CRISPR RNA DR sequence alignment of M. tuberculosis, M. canettii, and S. thermophilus displaying conservation of compatible bases involved in RNA stem-loop formation that may interact with Cas endoribonucleases (13, 23).

10.1128/mSystems.00934-20.7 FIG S2 Multiple sequence-structure alignment of CRISPR DR by LocARNA. Multiple sequence-structure alignment of CRISPR DR from CRISPRmap database calculated by LocARNA showing conserved structural motif involved in stem formation. Compatible base pairs are colored in similar hue. Dotted positions represent unpaired bases, and matching positions in parentheses represent paired bases. The bar graphs represent the degree of conservation of the alignment columns. Download FIG S2, TIF file, 2.8 MB. Copyright © 2021 Singh et al. 2021 Singh et al. This content is distributed under the terms of the Creative Commons Attribution 4.0 International license.

Further, to independently validate the source, we analyzed the global collection of Cas10 protein sequences obtained from a previous study (15) (Table S4c). Cas10 was chosen for comparative analysis since it is encoded by a signature *cas* gene of CRISPR type III system which forms the major part of the effector complex and interacts with the CRISPR repeat (15). On the basis of Cas10 phylogeny, we found that the MTBC clade belonging to phylum *Actinobacteria* clustered to a clade consisting of members of *Streptococcus* spp. and Lactobacillus ruminis both belonging to the phylum *Firmicutes* with high bootstrap support (Fig. 3b). The evolutionary proximity of MTBC Cas10 with its corresponding homologs in *Streptococcus* spp. is in line with the observation of conserved CRISPR repeats and independently supports our finding of *Streptococcus* like *Firmicutes* bacterium to probably serve as the source for HGT-acquired CRISPR-Cas type III-A system in MTBC. A recent study has demonstrated that M. tuberculosis type III-A CRISPR system utilize Cas10-activated cyclic hexa-adenylate (cA6) signaling to degrade invading RNA to enhance immunity. The phylum-wise comparison of characterized type III-A CRISPR systems revealed that the cA6-dependent signaling strategy is common between *Actinobacteria* members such as M. tuberculosis and *Firmicutes* members such as S. thermophilus, while other studied archaeal, *Deinococcus-Thermus*, and proteobacterial phyla utilize the cA4-modulated immunity (14). Although physical evidence of genetic exchange between *Mycobacterium* and *Streptococcus* is missing, interphylum HGT is a major evolutionary process and has been suggested to occur frequently for transfer of metabolic genes in many mesophilic bacteria (33). Thus, our results also strongly indicate that an HGT-driven acquisition of CRISPR-Cas type III-A system likely occurred in the last common ancestor of *M. canettii* (STB-A) and MTBC, from a *Streptococcus-*like environmental bacterium.

### Evolutionary role of IS*6110* transposition in the diversification of CRISPR-Cas system in M. tuberculosis lineages.

**FIG 4:**
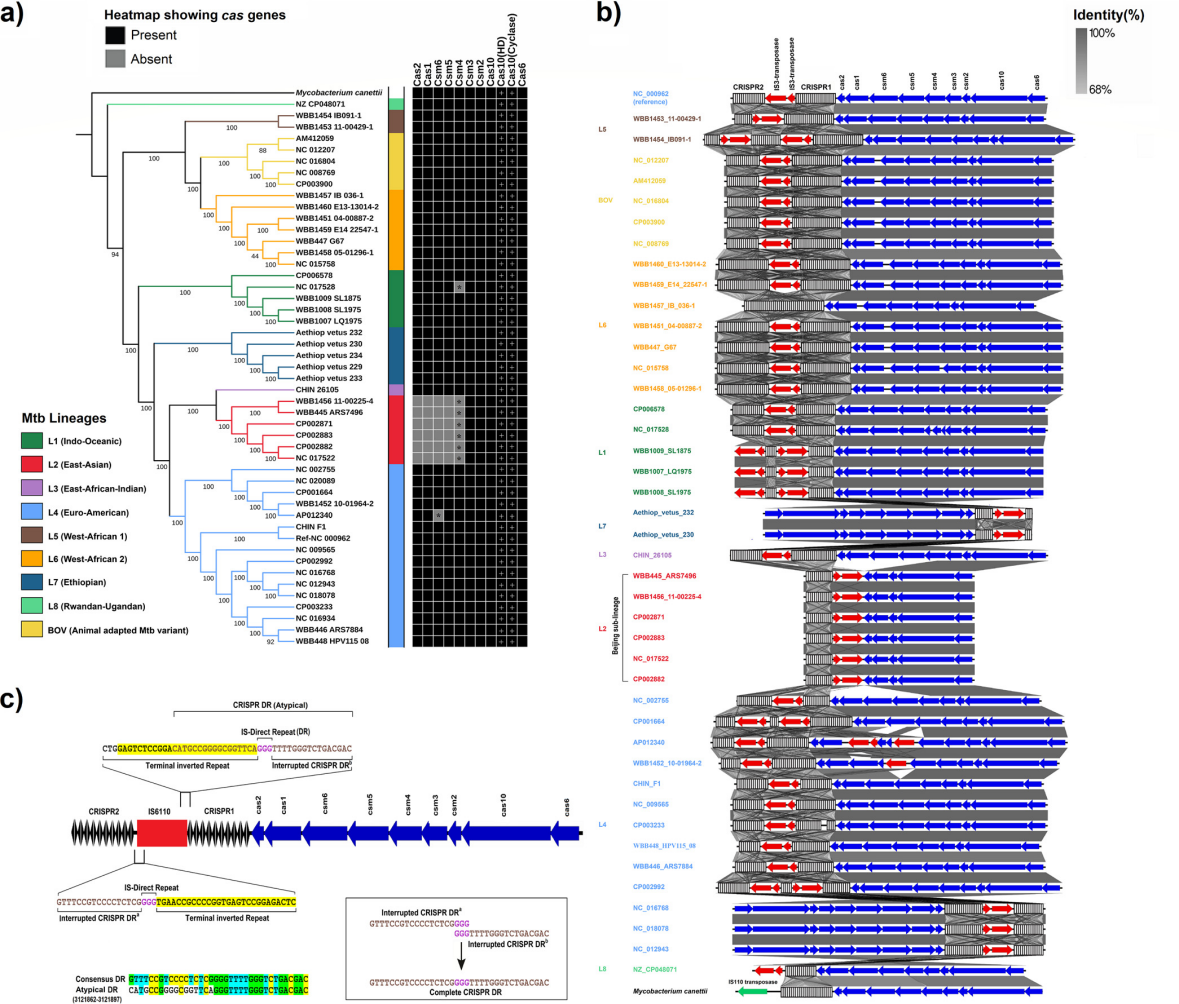
Comparison of genetic organization of the CRISPR-Cas region in M. tuberculosis lineages shows IS*6110* (IS3 family transposon) element-derived interruption. (a) Phylogeny of M. tuberculosis lineages showing differential *cas* gene distribution. Maximum likelihood phylogeny of 43 M. tuberculosis strains belonging to lineages 1 to 8 and five animal-adapted strains of M. tuberculosis variant bovis rooted with *M. canettii* as an outgroup was inferred from core genome SNPs. The different clades are color coded according to previously defined lineages (7). The comparison shows lineage-specific absence of certain *cas* genes in lineage 2 members (gray boxes), while truncation of gene is indicated with an asterisk inside the box. The plus sign indicates the presence of active sites of Cas10 HD and cyclase domain. (b) Pattern of IS*6110* transposition within the CRISPR-Cas locus in M. tuberculosis lineages. The CRISPR-Cas genomic clusters based upon BLASTN pairwise alignments are visualized as linear arrow comparison plots. The coding regions are represented by arrows, and CRISPR loci are shown as striated rectangular boxes. Blue arrows indicate *cas* genes, and red and green arrows represent different types of transposases, respectively. The arrow orientation represents forward/reverse positioning on the genome. The gray vertical blocks between sequences indicate regions of percent identity, shaded according to BLASTN results, and the degree of sequence identity is indicated by the intensity of gray color. Sequence names are color depicted according to previously defined lineages, as in panel a. Ancestral arrangement of the CRISPR-Cas system in the genome of *M. canettii* is represented at the bottom. (c) Schematic representation of M. tuberculosis CRISPR-Cas locus highlighting the overlap region between CRISPR1 and IS*6110*. The coding regions are shown as arrows and CRISPR loci as diamonds. Blue arrows indicate *cas* genes, and the red rectangle marks the relative position of IS*6110*. The flanking region of IS*6110* is magnified to show interruption of CRISPR repeat into two halves due to IS*6110* transposition. The CRISPR1 locus repeat at position 3121862 to 3121897 is an atypical repeat with overlap between a part of IS-terminal inverted repeat (IR) and half CRISPR direct repeat (DR). IR is highlighted in yellow. The CRISPR DR sequence is in brown text. The atypical overlapping region highlighted in yellow with brown text, and the DR site of IS*6110* is denoted by the text in purple. The alignment of this atypical CRISPR repeat with consensus repeat is shown at the bottom on left. Complete CRISPR DR retrieved by overlapping the interrupted CRISPR DR is shown inside the box.

To delve deeper into the evolution of CRISPR diversification, we carried out a whole-genome core single nucleotide polymorphism (SNP)-based phylogenetic analysis of M. tuberculosis lineages. We obtained the reference data sets for M. tuberculosis lineages from four independent studies by Coll et al. (34), Phelan et al. (35), Nebenzahl-Guimaraes et al. (36), and Ngabonziza et al. (7) (Table S5a). These phylogenetic lineages were further validated using the Snippy program for SNP calling and SNP-IT for lineage classification (37). Next, CRISPR-Cas loci were predicted using CRISPRCasFinder in all lineages. The signature *cas10* gene of the CRISPR type III system was conserved in all lineages. Since Cas10-mediated cA6 generation has been shown to be critical for M. tuberculosis CRISPR defense (14), we also looked for the conservation of active sites in the Cas10 cyclase domain that generates the cyclic oligoadenylates. The active sites (GGDD) of Cas10 cyclase domain were conserved in all M. tuberculosis lineages (Fig. 4a and Fig. S3a). While the *in vitro* DNA cleavage activity of the other critical domain of Cas10, HD nuclease domain, is still challenged (14), our results showed the conservation of active site residues (HD) in all lineages (Fig. 4a and Fig. S3b). Our results confirmed the absence of *csm4* (truncated), *csm5*, *csm6*, *cas1*, and *cas2* in a strain cluster belonging to members of Beijing lineage (sublineage of lineage 2) (Fig. 4a), consistent with previous reports (18, 38). Deletions in this region suggest compromised genome defense; nonetheless, few studies have reported mutations in *cas1* and *cas2* to affect drug resistance in bacteria and the ability to accumulate DNA mutations without affecting survival (39, 40). Therefore, it has been proposed that these deletions prove advantageous and probably better adapt the Beijing strain to infect humans and spread faster, despite compromising on its phage immunity (41). A frequent cause of genomic deletions in bacteria is related to the movement of mobile genetic elements and although insertion of IS*6110* in M. tuberculosis CRISPR-Cas locus has been observed (19), its impact on the CRISPR-Cas system is poorly understood. Apart from implication of IS*6110* transposition in host adaptation (19), studying differential insertion sites in diverse strains also has potential use as molecular markers for identifying strain-specific outbreaks, as seen with the Central Asia outbreak (CAO) clade, where a specific IS*6110* insertion was detected unique to this major epidemic clade of Beijing genotype (42).

10.1128/mSystems.00934-20.5 TABLE S5 M. tuberculosis lineages and BLASTN result for M. tuberculosis H37Rv undetected spacer sequence. (a) List of M. tuberculosis lineages used in the study and (b) BLASTN results of retrieved spacer sequence against MTBC spacer sequences. Download Table S5, XLSX file, 0.01 MB. Copyright © 2021 Singh et al. 2021 Singh et al. This content is distributed under the terms of the Creative Commons Attribution 4.0 International license.

10.1128/mSystems.00934-20.8 FIG S3 Multiple sequence alignment of Cas10 HD nuclease and cyclase domain. Multiple sequence alignment by MAFFT using default parameters showed conservation of active site residues marked with an asterisk in the cyclase domain (a) and HD nuclease domain (b) in all M. tuberculosis lineages and animal-adapted members. Download FIG S3, PDF file, 2.2 MB. Copyright © 2021 Singh et al. 2021 Singh et al. This content is distributed under the terms of the Creative Commons Attribution 4.0 International license.

Pairwise alignment of CRISPR-Cas loci was carried out for all M. tuberculosis lineages and *M. canettii* using BLASTN with M. tuberculosis H37Rv as the reference genome (Fig. 4b). While the typical organization of the CRISPR-Cas locus with a single copy of IS*6110* inserted in CRISPR DR, as present in the reference genome M. tuberculosis H37Rv (GenBank accession number NC_000962), is common to most other strains belonging to different lineages; distinct genomic variations in locus organization were observed in some isolates (Fig. 4b). As seen in Fig. 4a and consistent with previous findings (18), deletions in the CRISPR-Cas locus in the Beijing sublineage (lineage 2) was observed (Fig. 4b), which seems to be mediated by the transposition of IS*6110* in that genomic location. The evidence of active transposition is strengthened from the observation of other lineages; for example, in lineage 1, two isolates show the typical M. tuberculosis organization, while isolates WBB1007_LQ1975, WBB1008_SL1975, and WBB1009_SL1875 possess two copies of IS*6110* in reverse orientation, with one copy inserted just outside the CRISPR locus (Fig. 4b). Presence of two IS*6110* copies between three CRISPR arrays was noted in CP002992 (lineage 4), CP001664 (lineage 4), and WBB1454_IB091-1 (lineage 5). CP003233 (lineage 4) shows presence of only one IS*6110* copy and three CRISPR arrays. Since there is no trace of another IS*6110* copy between the proximal two arrays of CP003233 separated by a short genomic region (118 bp), we are unable to fully attribute IS*6110* as the cause of this disruption. Transposition of IS*6110* in the *cas* gene region of AP012340 (lineage 4) leaves a partial copy of IS*6110* with a single ORF encoding the transposase (apart from two complete copies) and results in disruption of *csm5* and truncation of *csm6* (Fig. 4b and a). A similar organization is seen in WBB1452_10-01964-2 (lineage 4), but *csm6* is conserved. The most unique observation was seen in a lineage 6 member, WBB1457_IB_036-1, that possesses a single long CRISPR array with no nearby IS*6110* insertion similar to *M. canettii*. Similarly, lineage 8 member NZ_CP048071 also has a single CRISPR array but a nearby copy of IS*6110* (Fig. 4b). These results suggest active transposition of IS*6110* in M. tuberculosis lineages has impacted evolution of CRISPR locus genomic region.

Therefore, we analyzed the classic CRISPR locus of M. tuberculosis H37Rv to better understand the mechanism of transposition. We observed that the IS*6110* IR overlaps with the DR of the CRISPR1 locus of the M. tuberculosis H37Rv reference genome (Fig. 4c). On the basis of genomic analysis, we propose that this overlapping repeat is a result of interruption of the CRISPR DR region by IS*6110*. The repeat at positions 3121862 to 3121897 is atypical, a combination of part of the 3′ IR of IS*6110* and a part of 5′ DR of CRISPR1. As expected, the other half of the CRISPR DR is present at the 5′ end of the IS*6110* on the forward strand (Fig. 4c). The 3-bp DR duplication generated by IS*6110* flanking to the point of insertion (43) is “GGG” in strain H37Rv (highlighted in purple in Fig. 4c). Next, we reconstructed the ancestral M. tuberculosis single array CRISPR locus by joining the two arrays seen in the present-day strain by tracing a previously undetected spacer sequence between the two loci. This spacer is not detected by commonly used identification tools such as CRISPRCasFinder and CRISPRDetect due to interruption of the repeat flanking the spacer sequence (Fig. S4). In order to validate this assumption, we performed BLASTN with the retrieved spacer sequence against all MTBC spacer sequences detected by CRISPRCasFinder. The results showed significant hit with 100% identity and coverage with spacer sequences from M. tuberculosis variant africanum, M. tuberculosis variant bovis, M. tuberculosis variant orygis, and M. tuberculosis variant caprae. This suggests yet again that the particular spacer sequence is MTBC specific and can theoretically join the two CRISPR arrays into a single continuous array locus, which was disrupted by IS*6110* during the course of evolution (Fig. S5 and Table S5b). Such unique spacer sequences, which have remained undetected by common tools due to interruption by IS*6110*, can have potential value in strain identification.

10.1128/mSystems.00934-20.9 FIG S4 Schematic representation of the M. tuberculosis CRISPR-Cas locus. The coding regions are shown as arrows, and CRISPR loci are shown as diamonds. The blue arrow indicates *cas* genes and the red rectangle marks the relative position of IS*6110*. CRISPR2-IS*6110* flanking region is magnified to show the 55-bp region containing undetectable spacer in green and partial CRISPR repeat in yellow. Download FIG S4, TIF file, 0.4 MB. Copyright © 2021 Singh et al. 2021 Singh et al. This content is distributed under the terms of the Creative Commons Attribution 4.0 International license.

10.1128/mSystems.00934-20.10 FIG S5 Reconstructed ancestral CRISPR locus in M. tuberculosis. Repeats are highlighted in yellow. The repeat where transposition occurred is highlighted in gray, and the site of insertion is denoted by an arrow. The spacer sequence that remains undetectable in CRISPRCasFinder and CRISPRDetect is highlighted in green. Download FIG S5, TIF file, 2.4 MB. Copyright © 2021 Singh et al. 2021 Singh et al. This content is distributed under the terms of the Creative Commons Attribution 4.0 International license.

On the basis of the results, we conclude that M. tuberculosis ancestor must have possessed only one long CRISPR array as seen in *M. canettii* (Fig. 4b). This array has since been interrupted by transposition of IS*6110*, which led to the formation of two CRISPR loci separated by IS*6110*, as seen in most present-day M. tuberculosis strains. Thus, our results show that the DR of CRISPR region acts as a hot spot for the insertion of IS*6110* during transposition, as previously suggested (44), and generates a “GGG” duplication at the M. tuberculosis H37Rv strain locus. Such active transpositions have impacted the evolution of CRISPR locus leading to genomic variations such as gene deletions and recombination. These variations may lead to emergence of new pathogenic properties, as exemplified by the Beijing lineage, which despite genomic losses utilizes a selective advantage for infection and has emerged as a better-adapted pathogen (45).

### Conclusion.

Genomic comparisons of M. tuberculosis with related bacteria offer valuable insights into the evolutionary history and emergence of pathogenic strains. The present study, a comprehensive comparative genomic analysis of 141 mycobacterial genomes, showed the exclusive presence of the CRISPR-Cas type III-A system in MTBC. Further analysis revealed that CRISPR-Cas type III-A system was likely acquired in the last common ancestor of STB-A and MTBC by a HGT-driven acquisition. The plausible source seems to be a *Streptococcus-*like environmental bacterium. Our work reveals that although the genomic organization of CRISPR-Cas locus is conserved in M. tuberculosis lineages, certain specific strains show considerable deletions. These deletions, best exemplified in Beijing sublineage members, are driven by active transposition of IS*6110*, which utilize the DR of CRISPR region for insertion. This work delineates the evolutionary events such as HGT and IS*6110*-driven genomic variations in mycobacteria to better comprehend the epidemiology of M. tuberculosis lineages.

## MATERIALS AND METHODS

### Genome sequences and CRISPR-Cas classification.

All available (141) genome sequences of *Mycobacteriaceae* covering five genera were downloaded from NCBI-RefSeq website on 24 August 2019. The genomic data and annotations were obtained from NCBI-FTP (ftp://ftp.ncbi.nlm.nih.gov/genomes/refseq/bacteria/). CRISPR loci were predicted using CRISPRCasFinder (https://crisprcas.i2bc.paris-saclay.fr/) with default parameters (22). CRISPRCasFinder comprises of a rating system based on several features. Short candidate arrays made up of one to three spacers often do not correspond to real CRISPRs and are therefore given the lowest evidence level, level 1. Evidence levels 2 to 4 are attributed on the basis of the combined degrees of similarity of repeats and spacers. Arrays with evidence level 1 or 2 indicate potentially false-positive results and were not considered for our analysis. Additionally, all predicted loci were manually checked, and those located in coding regions were discarded. CRISPRmap (v1.3.0-2013) (23) was used to provide conserved motifs, family, and superclass based on structural and sequence similarities.

Cas proteins were identified using CRISPRCasFinder and HMMER 3 (46) against a collection of 395 Cas protein profiles obtained from a previous study (15). *cas* genes were annotated and naming of *cas* genes, and their classification into types and subtypes was carried out as described by Makarova et al. (15). Cas proteins were also cross-verified from the respective NCBI genome annotations.

### 16S rRNA gene sequence-based phylogenetic tree construction.

16S rRNA gene-based comparative phylogenetic analysis was performed for all downloaded genomes. 16S rRNA sequences were obtained from the NCBI genome annotation files of the downloaded genomes (downloaded on 24 August 2019; see Table S1a in the supplemental material). To create the tree, multiple sequence alignment of the 16S rRNA gene sequences corresponding to the 141 gene copies were performed by MAFFT v7 using default parameters (47). The alignment was used to compute a maximum likelihood phylogenetic tree using the GTR+G model in RAxML-NG v1.0.1 (48), and branch support was computed with 1,000 bootstrap replicates. The tree was midpoint rooted and visualized by iTOL software (https://itol.embl.de/).

### MTBC CRISPR spacer target identification and relatedness.

All available complete genomes of phages and plasmids were downloaded from the NCBI ftp server on 7 July 2020. Redundant genomes were removed, and a database was constructed using the NCBI-BLAST+ 2.9.0 command-line tool. BLASTN was performed for all CRISPRCasFinder-identified spacers against NCBI phage and plasmid databases, with 90% identity and query coverage. Significant matches were summarized in bipartite networks with edges between spacers and their targets and visualized using the Cytoscape software (49). Edges between network nodes were assigned when a protospacer matching a spacer in a given host was identified in a phage.

CRISPRStudio (50) was used to visualize the CRISPR locus using default parameters; it compares spacer sequences present in a CRISPR array and then clusters them based on sequence similarities. To identify unique spacers at default settings, it considers spacer pairs with ≤2 mismatches as identical. CRISPRStudio requires gff3 file format as an input generated by CRISPRDetect. CRISPR loci common to both CRISPRCasFinder and CRISPRDetect (51) were used for visualization by CRISPRStudio.

### STB phylogeny and sequence analysis.

All available STB strains were downloaded from NCBI-RefSeq website on 24 August 2019. Core genome alignment was done using Roary v3.13.0 for all the genomes (52). Alignment also included genomes of M. tuberculosis H37RV (GenBank accession number NC_000962), M. tuberculosis RW-TB008 (accession number NZ_CP048071), M. tuberculosis variant bovis (accession number NC_016804.1), and NTMs (M. kansasii [accession number NZ_CP019888.1] and M. marinum [accession number NZ_HG917972]). A maximum likelihood phylogenetic tree was constructed using the GTR+G model in RAxML-NG v1.0.1, and branch support was computed with 1,000 bootstrap replicates, using M. marinum as an outgroup. The tree was visualized using iTOL.

### Genomic island prediction.

Genomic islands (GIs) were identified and analyzed by IslandViewer 4 (31), which integrates four different and accurate GI predictor tools: IslandPath-DIMOB, SIGI-HMM, IslandPick, and Islander. IslandViewer was used to analyze GIs in STB-A (GenBank accession number NC_015848.1) and M. tuberculosis genome (accession number NC_018143.2).

### Phylogenetic analysis of CRISPR repeats and Cas10 families.

Consensus CRISPR repeat sequence of MTBC and *M. canettii* genomes was used as a query sequence against CRISPRmap repeat database (v1.3.0-2013). CRISPRmap program constructs a hierarchical cluster tree based on multiple sequence-structure alignment of repeat sequences and the minimum free energy (MFE) structures generated by LocARNA to find relatedness (53) and provide a consensus MFE structure. A separate alignment of CRISPR DR RNA sequence of M. tuberculosis, *M. canettii*, and S. thermophilus was performed by T-COFFEE at default parameters (https://www.ebi.ac.uk/Tools/msa/tcoffee/). The sequence logo of CRISPR repeats of aligned family members was obtained using WebLogo (54).

To find the closest relative of M. tuberculosis Cas10, the global collection of Cas10 sequence data described in a study by Makarova et al. (15), was downloaded from the Batch Entrez website (https://www.ncbi.nlm.nih.gov/sites/batchentrez). Multiple sequence alignments were performed to align closely related sequences by MAFFT v7, and it was also used to merge these alignments. The phylogenetic tree was reconstructed using LG+G model in the IQTREE v1.6.2 (55). The same program was used for 1,000 bootstrap calculation. Phylum level classification was done manually with the help of the NCBI-Taxonomy browser (https://www.ncbi.nlm.nih.gov/Taxonomy/Browser/wwwtax.cgi) for tree annotation. The tree was midpoint rooted and visualized using iTOL.

### Whole-genome core SNP phylogeny of M. tuberculosis lineages and CRISPR-Cas loci prediction.

We downloaded M. tuberculosis lineages (lineages 1 to 8 and animal-adapted lineages) as reference data sets comprising of 48 genomes from three different studies, namely, 24 genomes from the study of Coll et al. (34), 18 genome sequences from Phelan et al. (35), five genome sequences from Nebenzahl-Guimaraes et al. (36) and one genome sequnece from Ngabonziza et al. (7) (Table S5a). *M. canettii* (GenBank accession number NC_015848.1) was used as an outgroup. Core genome SNP calling was done using Snippy v4.4 (https://github.com/tseemann/snippy) using M. tuberculosis H37Rv as the reference genome. SNP-IT v1.1 (37) program was also used to predict and confirm M. tuberculosis lineages. Core genome SNP alignment file was used to compute a maximum likelihood phylogenetic tree using the GTR+G+ASC_LEWIS model in RAxML-NG v1.0.1 (48). Branch support was computed with 1,000 bootstrap replicates. The tree was visualized using the iTOL server. The CRISPR-Cas locus was predicted using CRISPRCasFinder. The presence of Cas10 HD and cyclase domains was predicted using Scanprosite (https://prosite.expasy.org/scanprosite/) and Cas10 HD and cyclase domains were aligned using MAFFT v7.

### IS element annotation within the CRISPR-Cas region.

CRISPR-Cas loci were predicted using the CRISPRCasFinder, as described earlier. The loci were extracted from the genomes using in-house perl script. Two sequences (NC_020089 and NC_016934) were removed due to assembly gaps in the CRISPR repeat region. IS*6110* transposase was annotated using Prokka v1.14 (56) in the CRISPR-Cas locus genome segment and aligned using NCBI-BLASTN using default parameters. Easyfig v2.2.3 was used to map and compare the CRISPR-Cas loci among M. tuberculosis lineage genomes. M. tuberculosis genome (accession NC_000962) was considered the reference genome, and *M. canettii* (accession NC_015848) was considered the ancestral genome. For identification of IS*6110* insertion at CRISPR loci, M. tuberculosis (accession number NC_000962) complete CRISPR-Cas locus sequence was extracted using in-house perl script. Overlapping region between IS*6110* and CRISPR repeats was analyzed and represented with CRISPR-Cas locus visualization generated using Easyfig v2.2.3 (57). We manually extracted genome sequence present between CRISPR2 and IS*6110* to identify a potential spacer sequence, as CRISPRCasFinder and CRISPRDetect were unable to detect it due to the split in the flanking CRISPR repeat. Further, BLASTN was performed with the retrieved sequence as a query against all MTBC spacer sequences using default parameters.
