# Supplementary material for: Comparative Genomic Analysis of Mycobacteriaceae Reveals Horizontal Gene Transfer-Mediated Evolution of the CRISPR-Cas System in the Mycobacterium tuberculosis Complex
Source: mSystems. 2021 Jan 19;6(1):e00934-20. doi: 10.1128/mSystems.00934-20 (PMC7820667; doi:10.1128/mSystems.00934-20)
Supplement: TABLE S2 [file mSystems.00934-20-st002.docx]

**Supplementary Table S2**: CRISPRmap results showing motifs, family and superclass of CRISPR repeats of *Mycobacterium* genus

| **Organism short ID** | **Organism name** | **Repeat** | **Motif** | **Famliy** | **Super class** | **Weblogo** | **Structure** |
| --- | --- | --- | --- | --- | --- | --- | --- |
| ELON_CR1 | *Mycolicibacter* *longobardus* strain DSM 45394 | GCCTCAACGAAAGCGCCGCGGCCGAAACCGCGGCGGG | motif 26 | - | E | - | 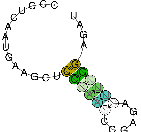 |
| FAUS_CR1 | *Mycolicibacterium* *austroafricanum* strain DSM 44191 | GGATCAATCCCCGCTTGCGCGGGGGAGAC | - | family 2 | B | 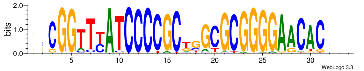 | - |
| FAUS_CR2 | *Mycolicibacterium* *austroafricanum* strain DSM 44191 | GTTTCCCCCGCGTAGGCGGGGATTGATC | - | family 4 | C | 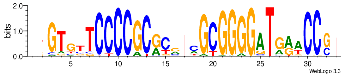 | - |
| FAUS_CR3 | *Mycolicibacterium* *austroafricanum* strain DSM 44191 | CCCCGCGTAGGCGGGGATTGATC | motif 22 | - | F | - | 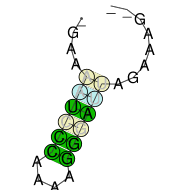 |
| FHAS_CR1 | *Mycolicibacterium* *hassiacum* DSM 44199 A3G5DRAFT | GTGATCTCCGTCCCTGAGACGGAGCCGCATTGCAGC | motif 19 | - | - | - | 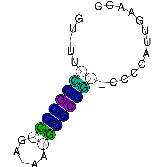 |
| MAF_CR1 | *Mycobacterium* *africanum* GM041182 | GTCGTCAGACCCAAAACCCCGAGAGGGGACGGAAAC | motif 5 | family 12 | E | 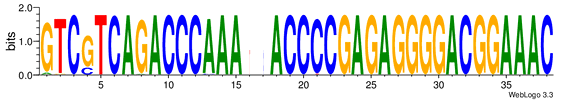 | 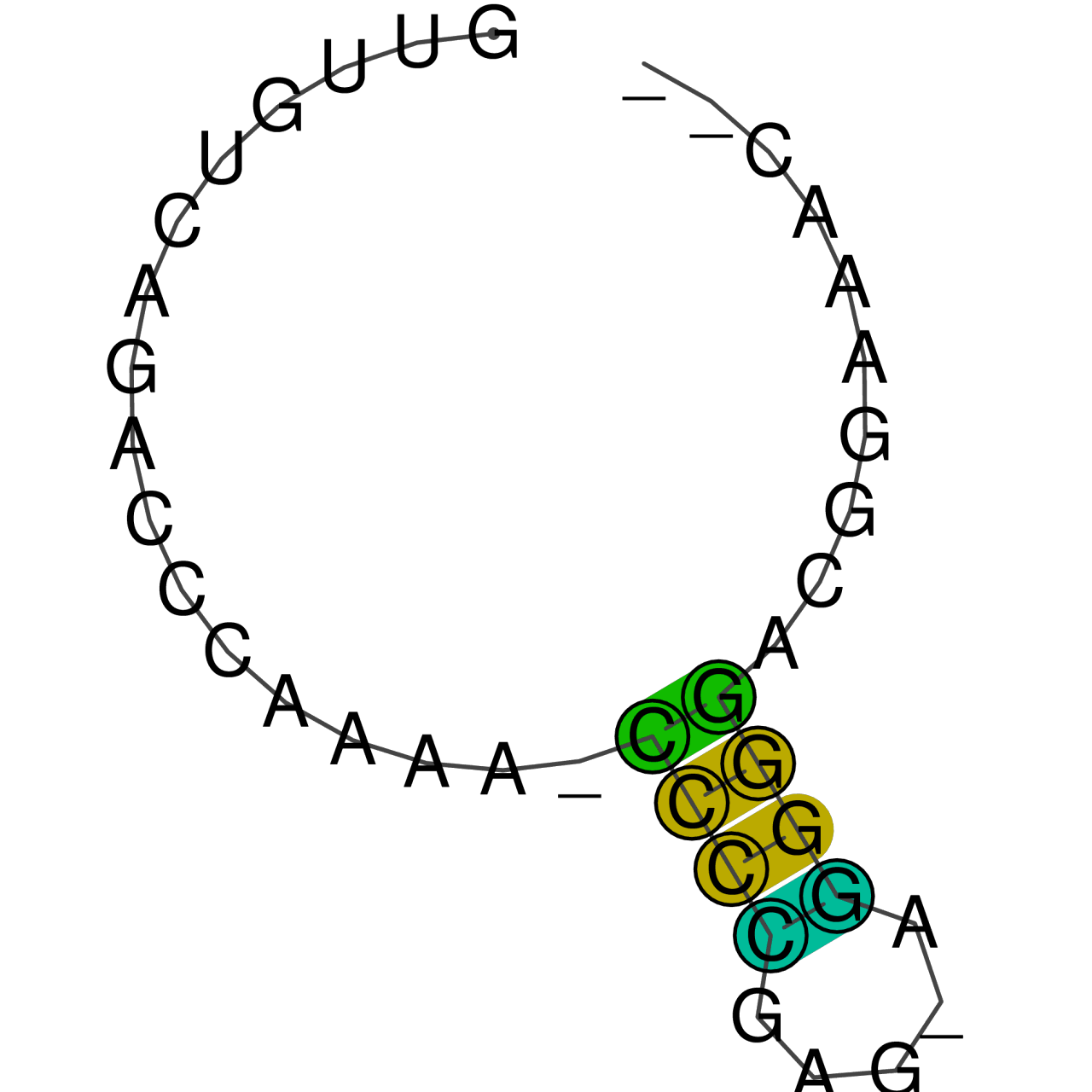 |
| MAF_CR2 | *Mycobacterium africanum* GM041182 | GTCGTCAGACCCAAAACCCCGAGAGGGGACGGAAAC | motif 5 | family 12 | E | 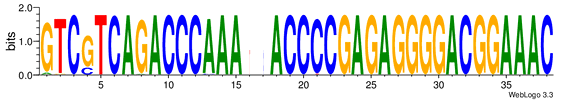 | 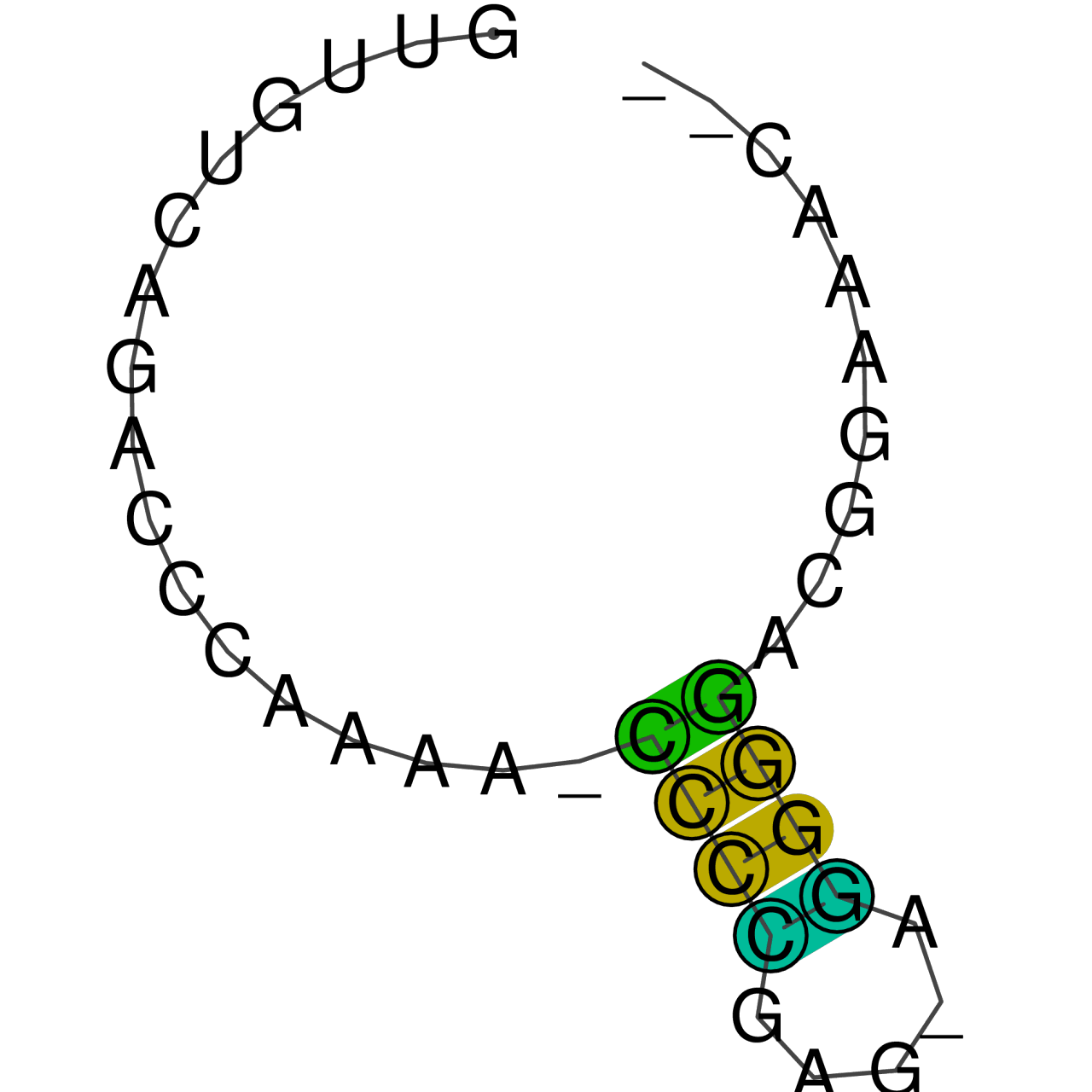 |
| MAV_CR1 | *Mycobacterium avium* 104 | TGCTCCCCGCGCAAGCGGGGATGA | motif 2 | family 4 | F | 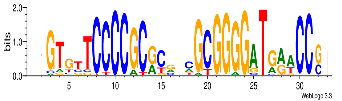 | 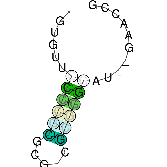 |
| MBOV_CR1 | *Mycobacterium bovis* AF2122/97 | GTCGTCAGACCCAAAACCCCGAGAGGGGACGGAAAC | motif 5 | family 12 | E | 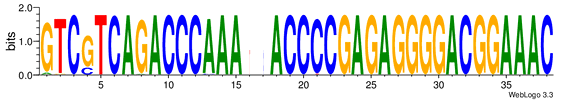 | 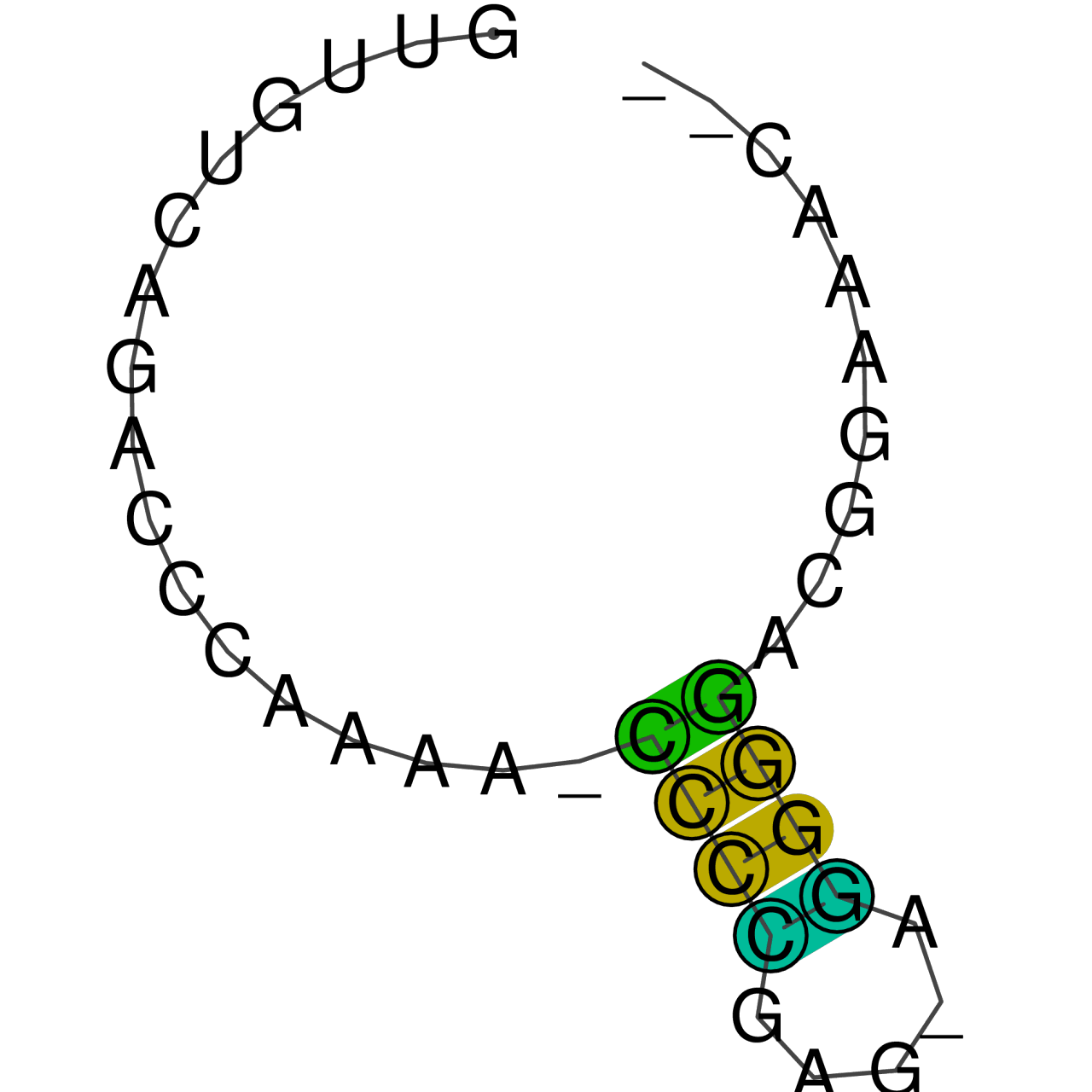 |
| MBOV_CR2 | *Mycobacterium bovis* AF2122/97 | GTCGTCAGACCCAAAACCCCGAGAGGGGACGGAAAC | motif 5 | family 12 | E | 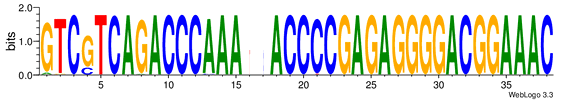 | 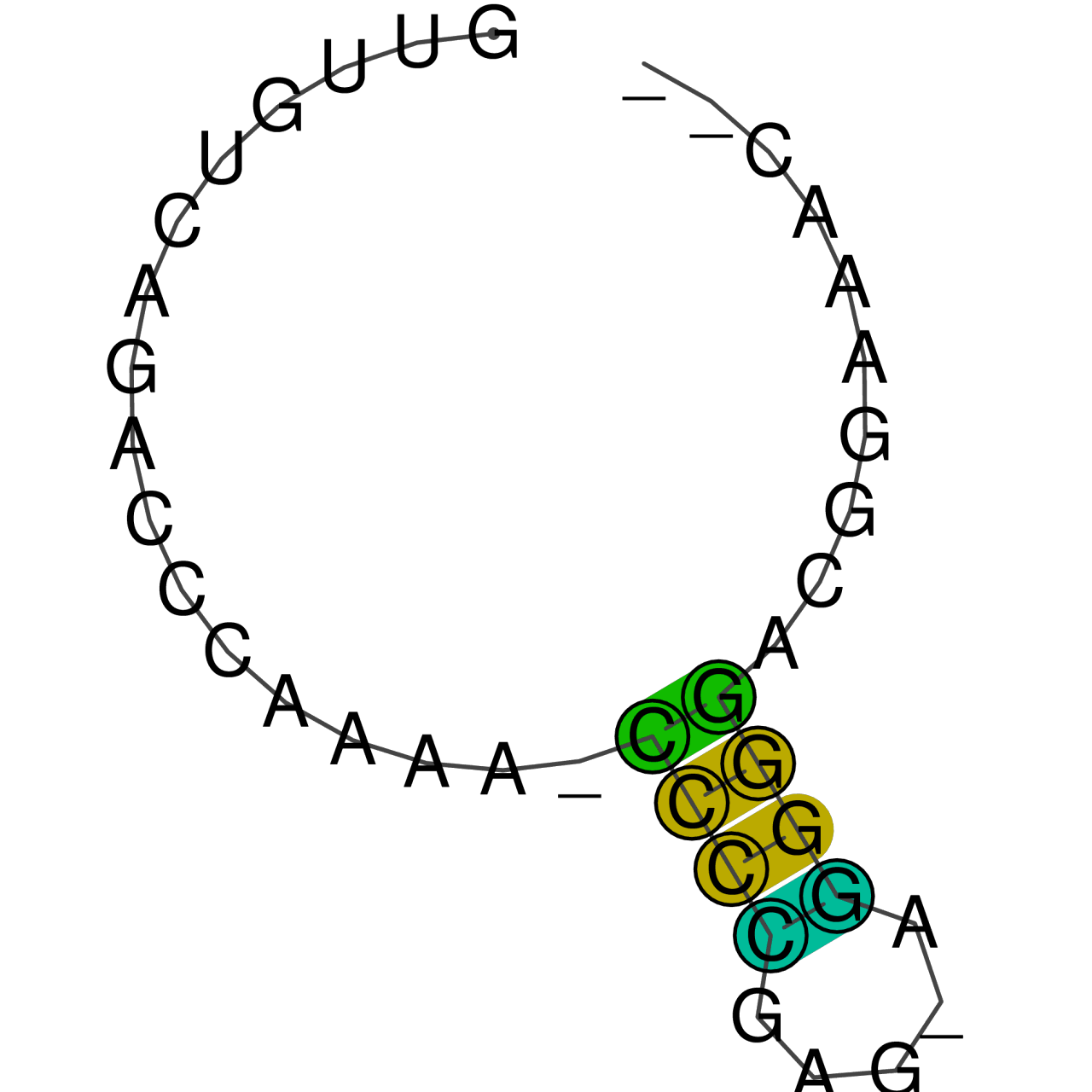 |
| MCAN_CR1 | *Mycobacterium canettii* CIPT 140010059 | GTCGTCAGACCCAAAACCCCGAGAGGGGACGGAAAC | motif 5 | family 12 | E | 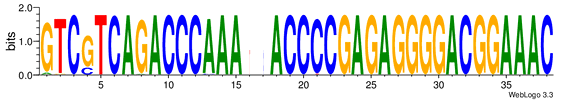 | 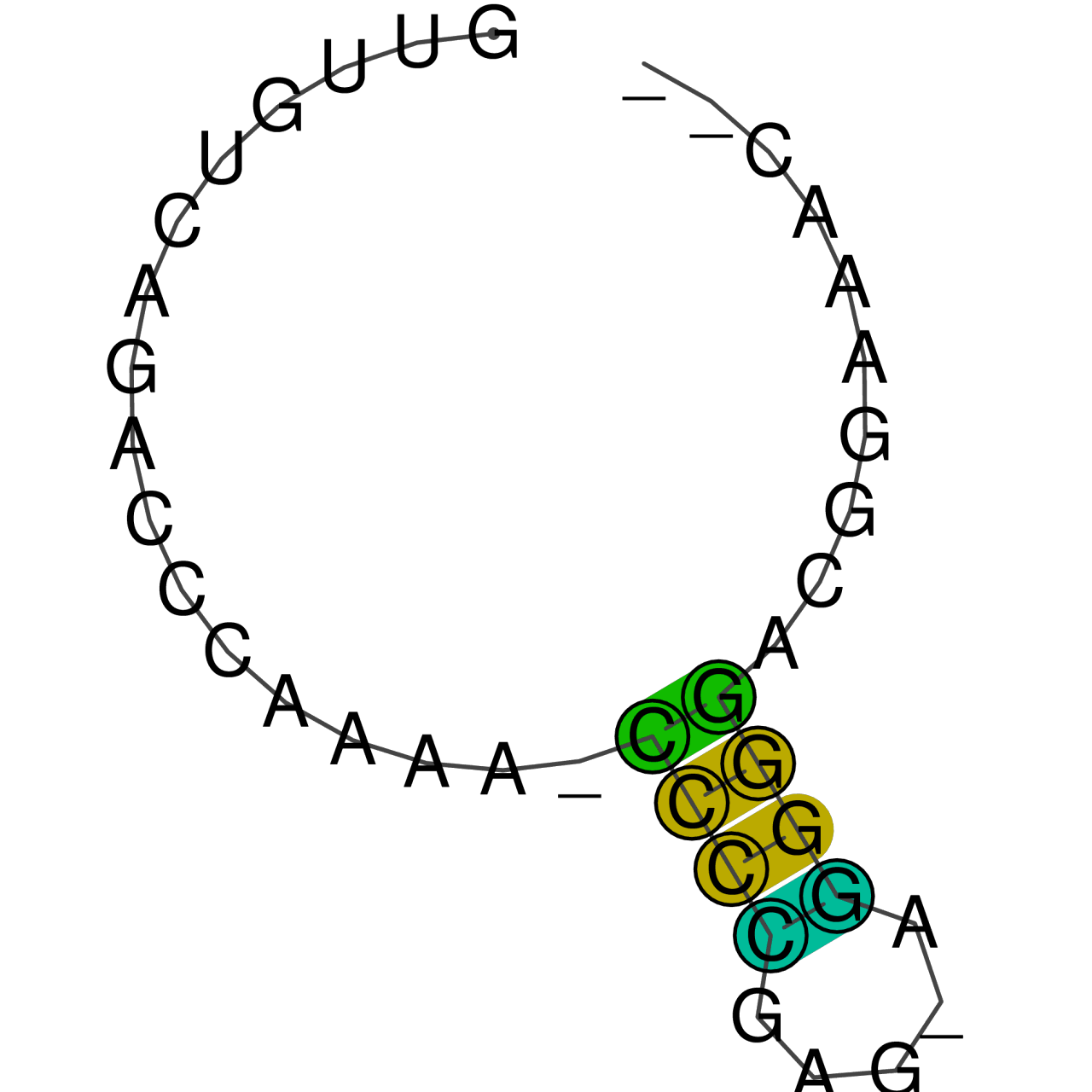 |
| MCAP_CR1 | *Mycobacterium caprae* strain Allgaeu genome | GTCGTCAGACCCAAAACCCCGAGAGGGGACGGAAAC | motif 5 | family 12 | E | 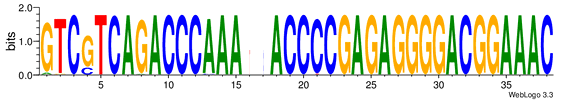 | 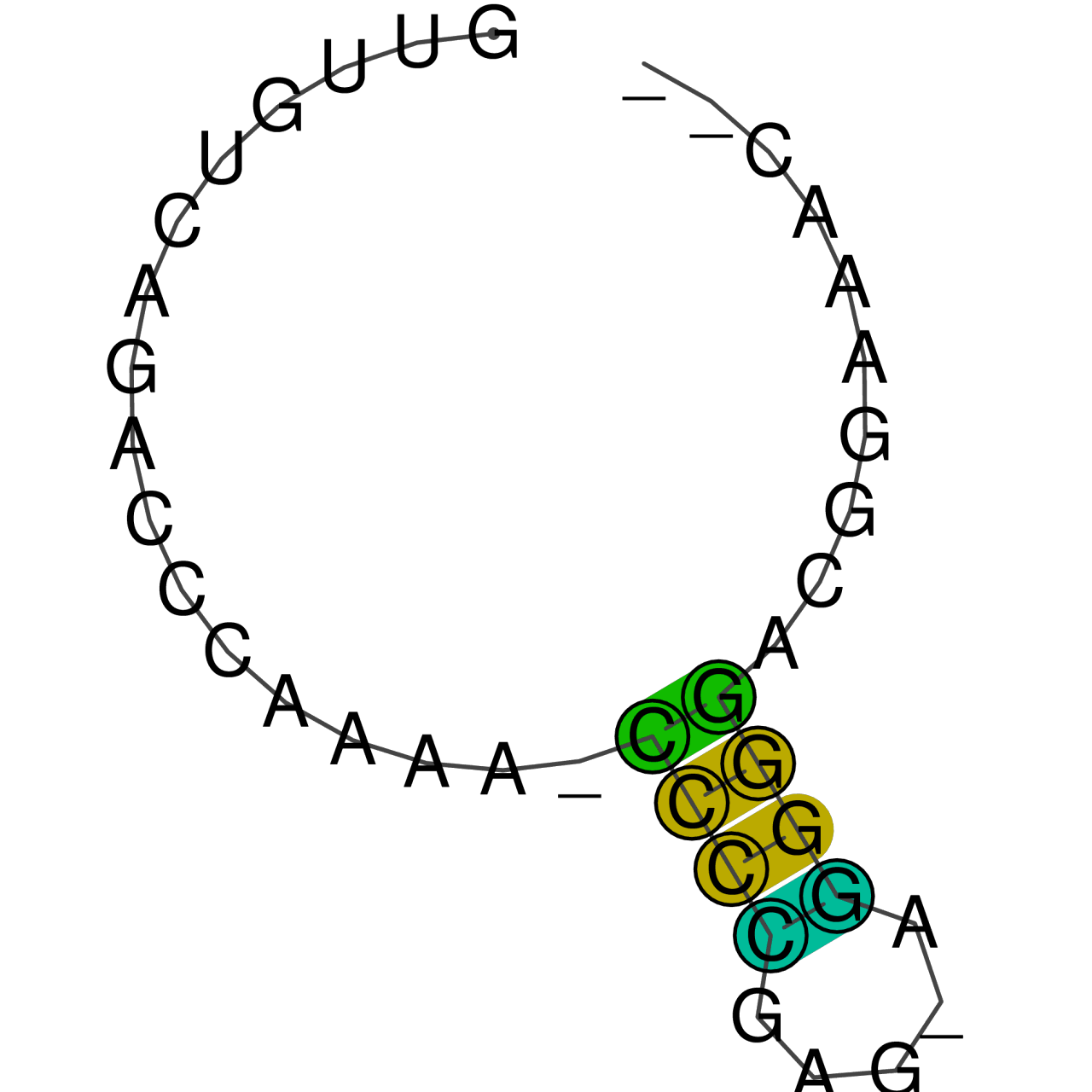 |
| MCAP_CR2 | *Mycobacterium caprae* strain Allgaeu genome | GTTTCCGTCCCCTCTCGGGGTTTTGGGTCTGACGACT | motif 30 | - | D | - | 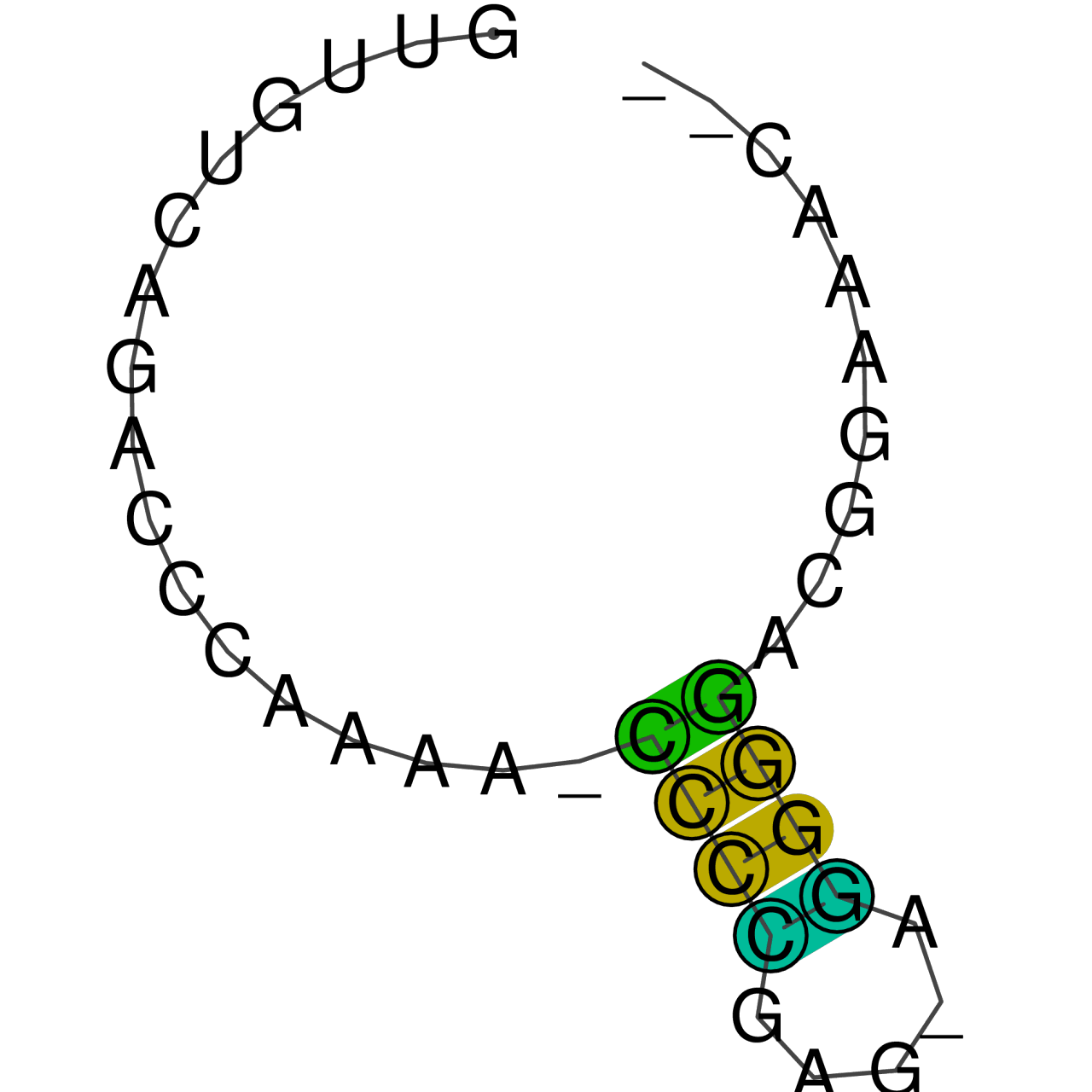 |
| MCEL_CR1 | *Mycobacterium celatum* strain ATCC 51131 | GTGCTCCCCGCGAATGCGGGGATGATCC | - | family 4 | C | 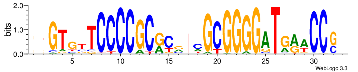 | - |
| MCEL_CR2 | *Mycobacterium celatum* strain ATCC 51131 | GGATCATCCCCGCATTCGCGGGGAGCAC | - | family 2 | B | 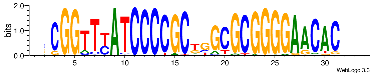 | - |
| MGAS_CR1 | *Mycobacterium gastri* 'Wayne' | GTTATTCCGGTCATTTTGGCCGGACACCATTGAGGC | motif 19 | - | - | - | 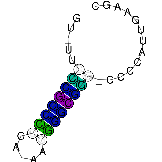 |
| MGAS_CR2 | *Mycobacterium gastri* 'Wayne' | GCCTCAATGGTGTCCGGCCAAAATGACCGGAATAAC | - | - | E | - | - |
| MGAS_CR3 | *Mycobacterium gastri* 'Wayne' | GTTATTCCGGTCATTTTGGCCGGACACCATTGAGGC | motif 19 | - | - | - | 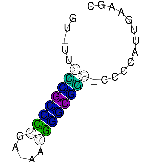 |
| MHEC_CR1 | *Mycobacterium* *heckeshornense* strain RLE Mheck | GCCGCAATGGAGCCCAGGCGAAATCACCTGGGAGAT | - | - | E | - | - |
| MHEC_CR2 | *Mycobacterium* *heckeshornense* strain RLE Mheck | ATCTCCCAGGTGATTTCGCCTGGGCTCCATTGCGGC | motif 19 | - | D | - | 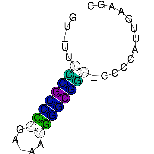 |
| MLAC_CR1 | *Mycobacterium* *lacus* strain DSM 44577 | GGATCACCCCCGCGTTTGCGGGGAGCAC | - | family 18 | B | 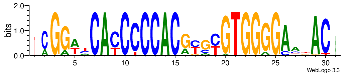 | - |
| MLAC_CR2 | *Mycobacterium* *lacus* strain DSM 44577 | GTGCTCCCCGCAAACGCGGGGGTGATCC | - | family 4 | C | 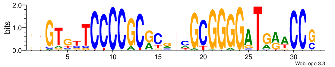 | - |
| MMIC_CR1 | *Mycobacterium* *tuberculosis* variant microti strain ATCC 19422 | GTCGTCAGACCCAAAACCCCGAGAGGGGACGGAAAC | motif 5 | family 12 | E | -  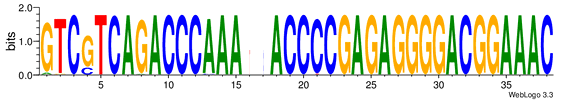 | 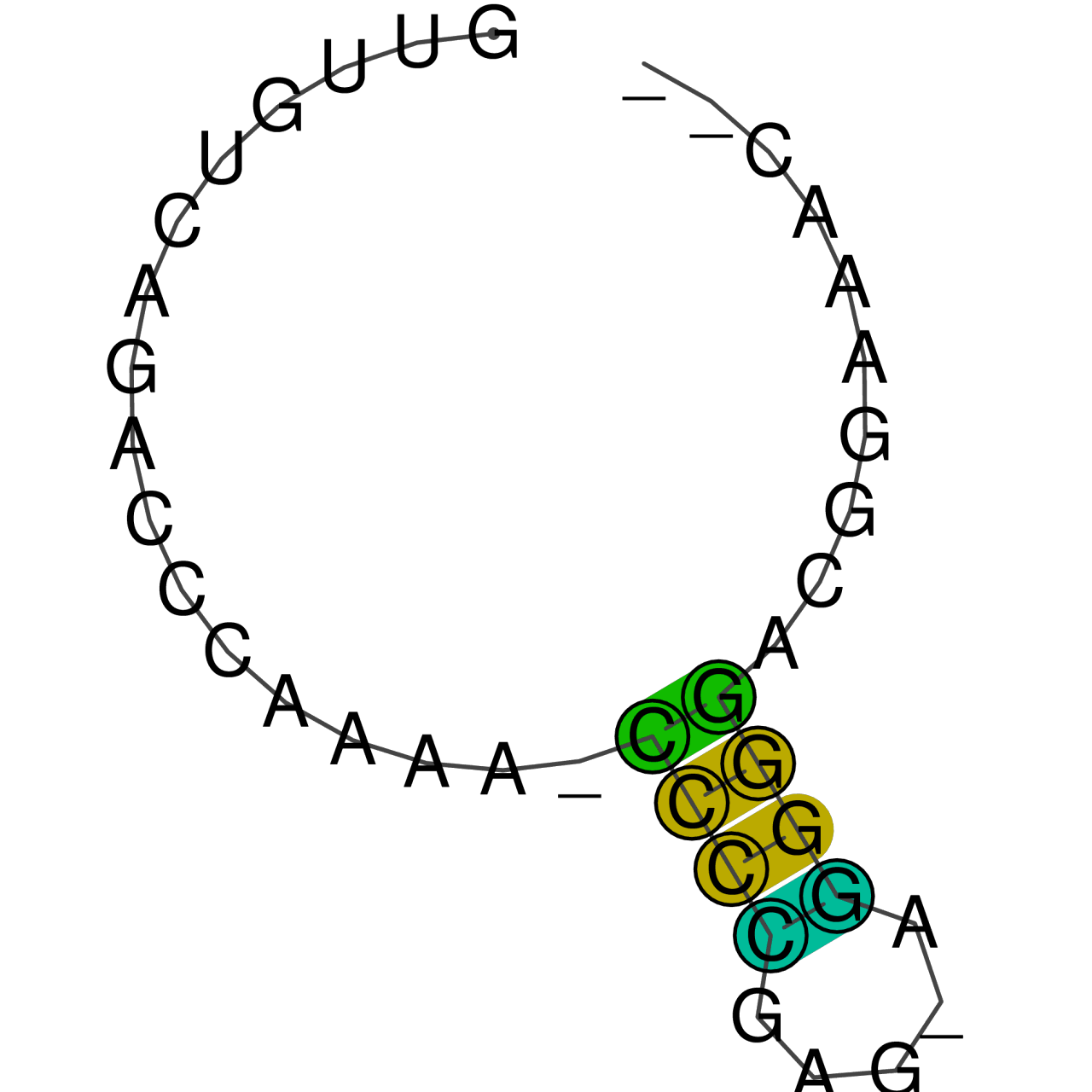 |
| MMIC_CR2 | *Mycobacterium* *tuberculosis* variant microti strain ATCC 19422 | TCAGACCCAAAACCCCGAGAGGGGACGGAAAC | motif 5 | family 12 | E | 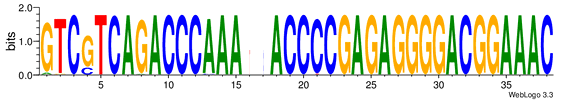 | 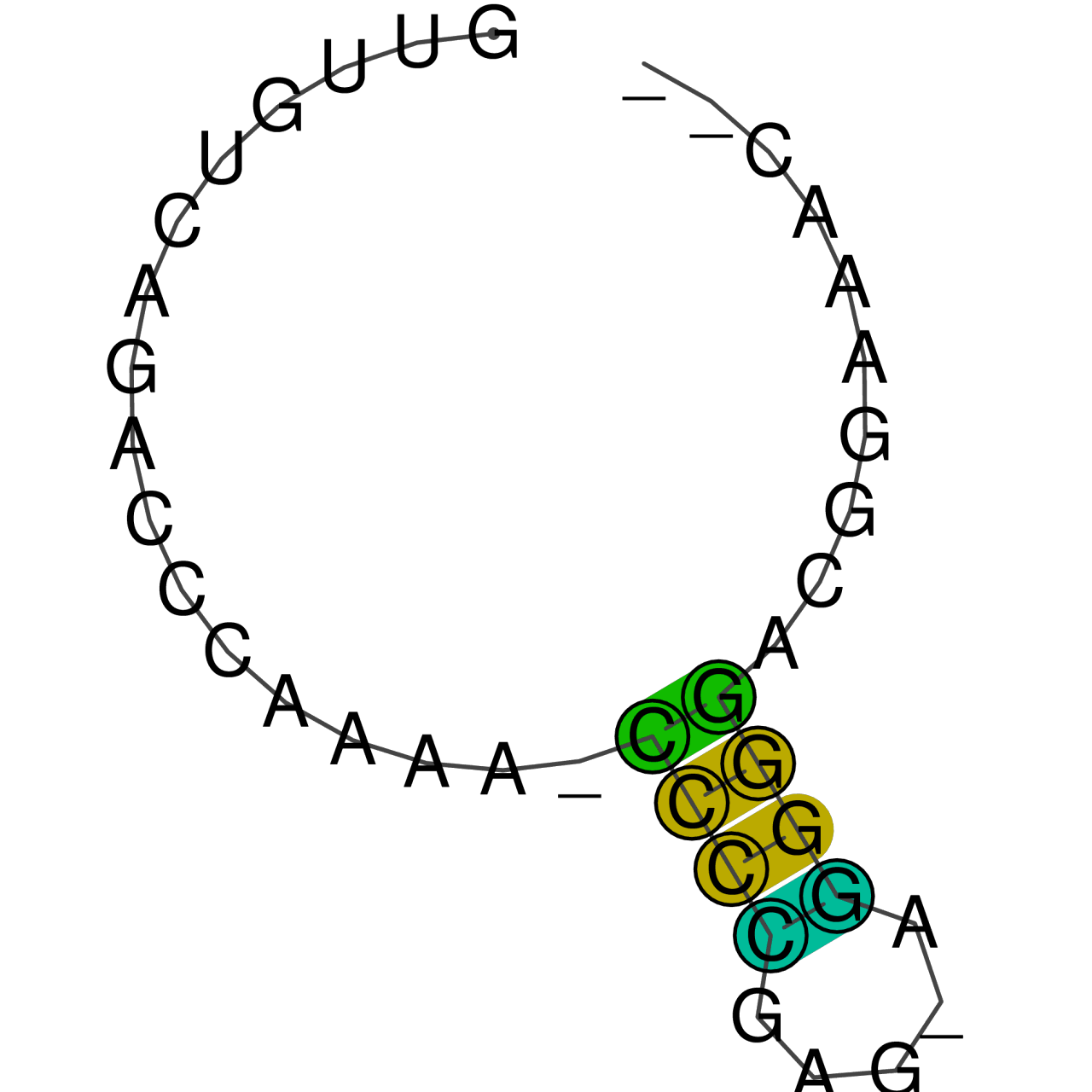 |
| MMUN_CR1 | *Mycobacterium* *mungi* strain BM22813 | GTCGTCAGACCCAAAACCCCGAGAGGGGACGGAAAC | motif 5 | family 12 | E | 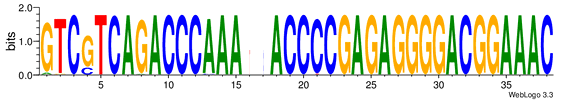 | 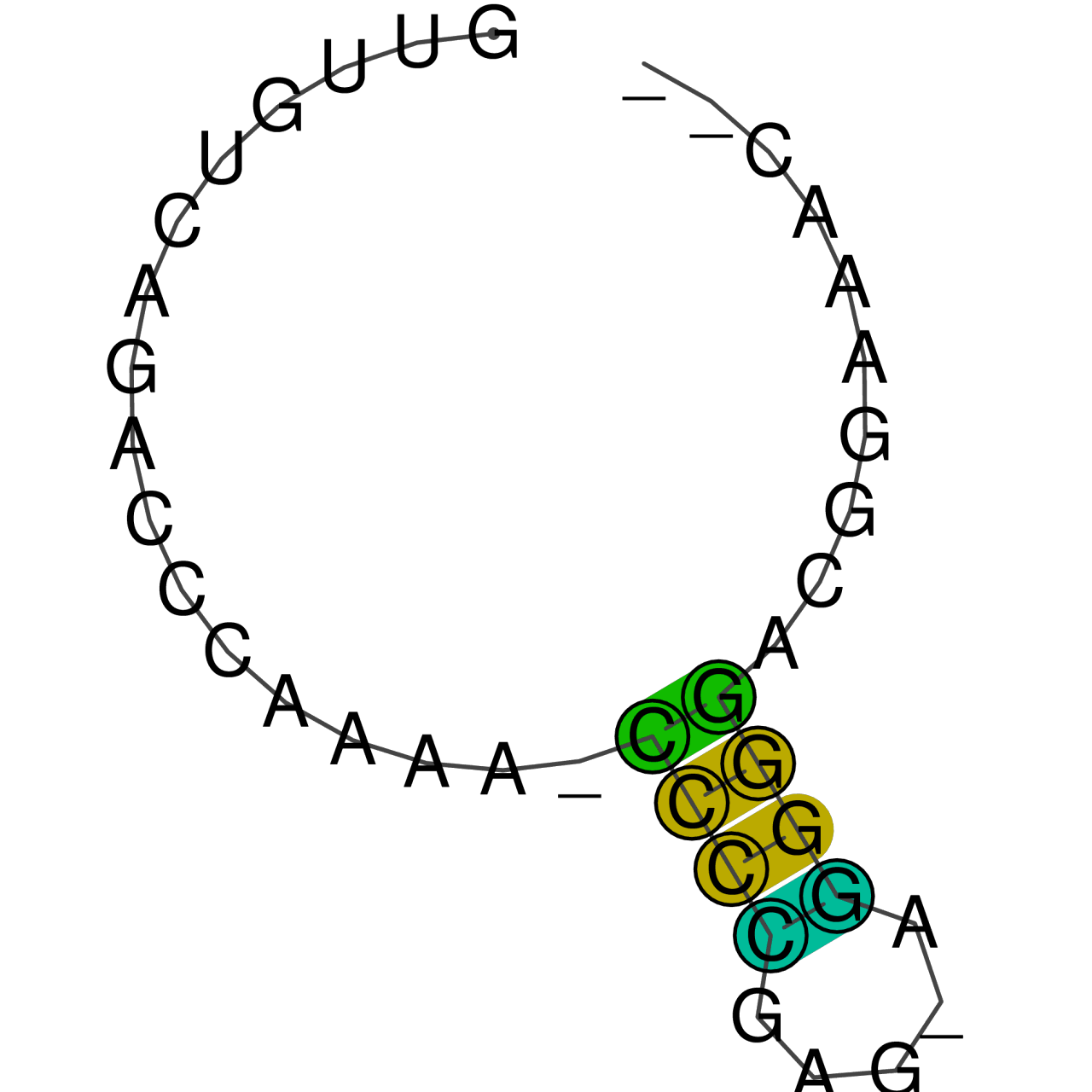 |
| MORY_CR1 | *Mycobacterium* *orygis* 112400015 Mo_54 | GTCGTCAGACCCAAAACCCCGAGAGGGGACGGAAAC | motif 5 | family 12 | E | 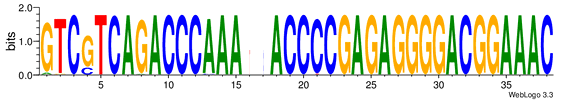 | 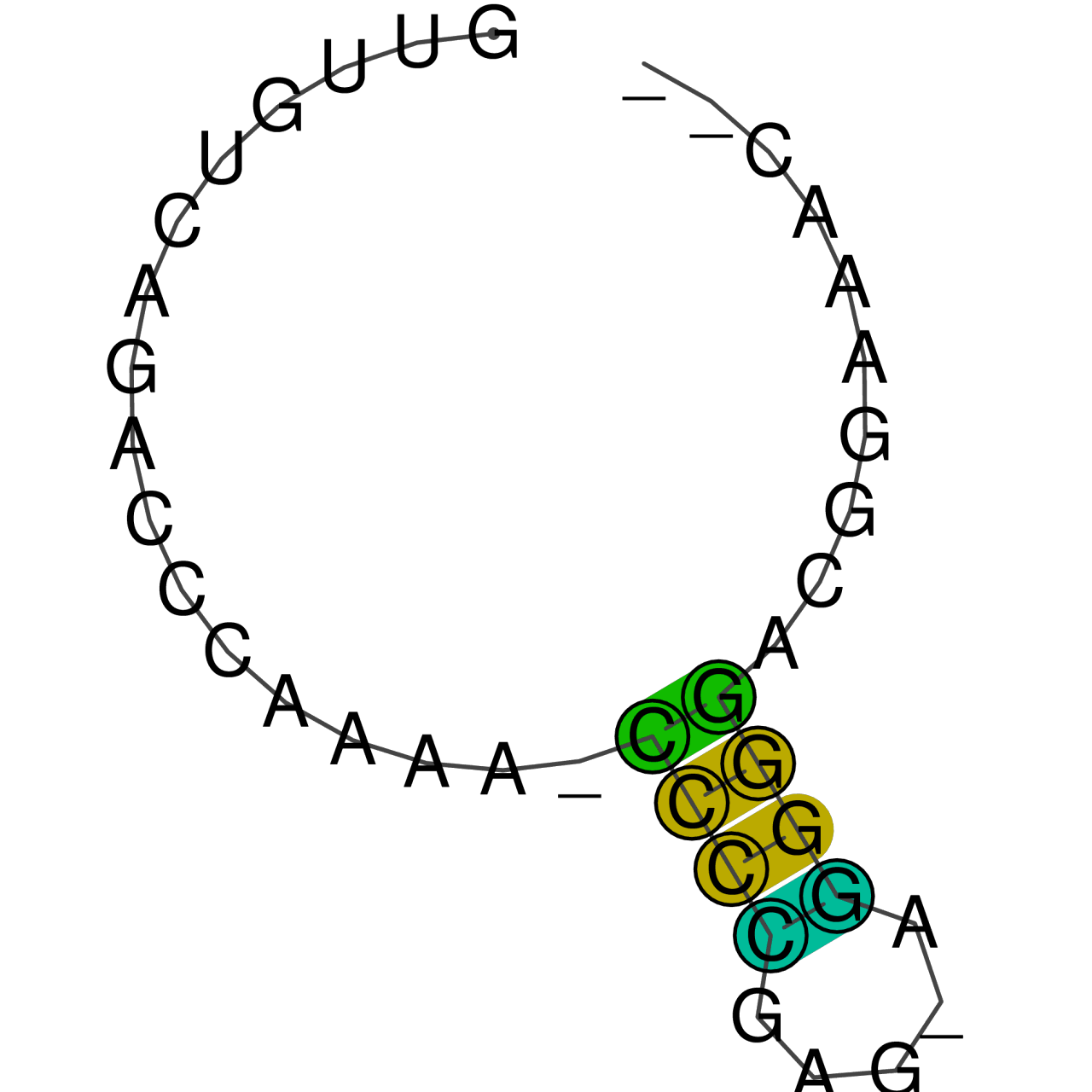 |
| MSHINJ_CR1 | *Mycobacterium* *shinjukuense* strain CCUG 53584 | GCGGCGGCCGTTTCGGCGGCCGCACTTCATCGAGGC | motif 19 | - | D | - | 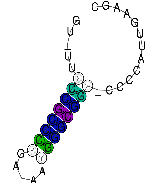 |
| MSHINJ_CR2 | *Mycobacterium* *shinjukuense* strain CCUG 53584 | GCCTCGATGAAGTGCGGCCGCCGAAACGGCCGCCGC | motif 26 | - | E | - | 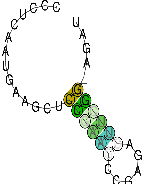 |
| MTUB_CR1 | *Mycobacterium* *tuberculosis* H37Rv | GTCGTCAGACCCAAAACCCCGAGAGGGGACGGAAAC | motif 5 | family 12 | E | 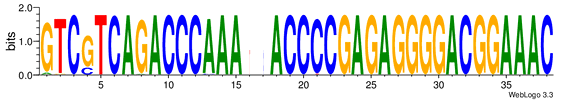 | 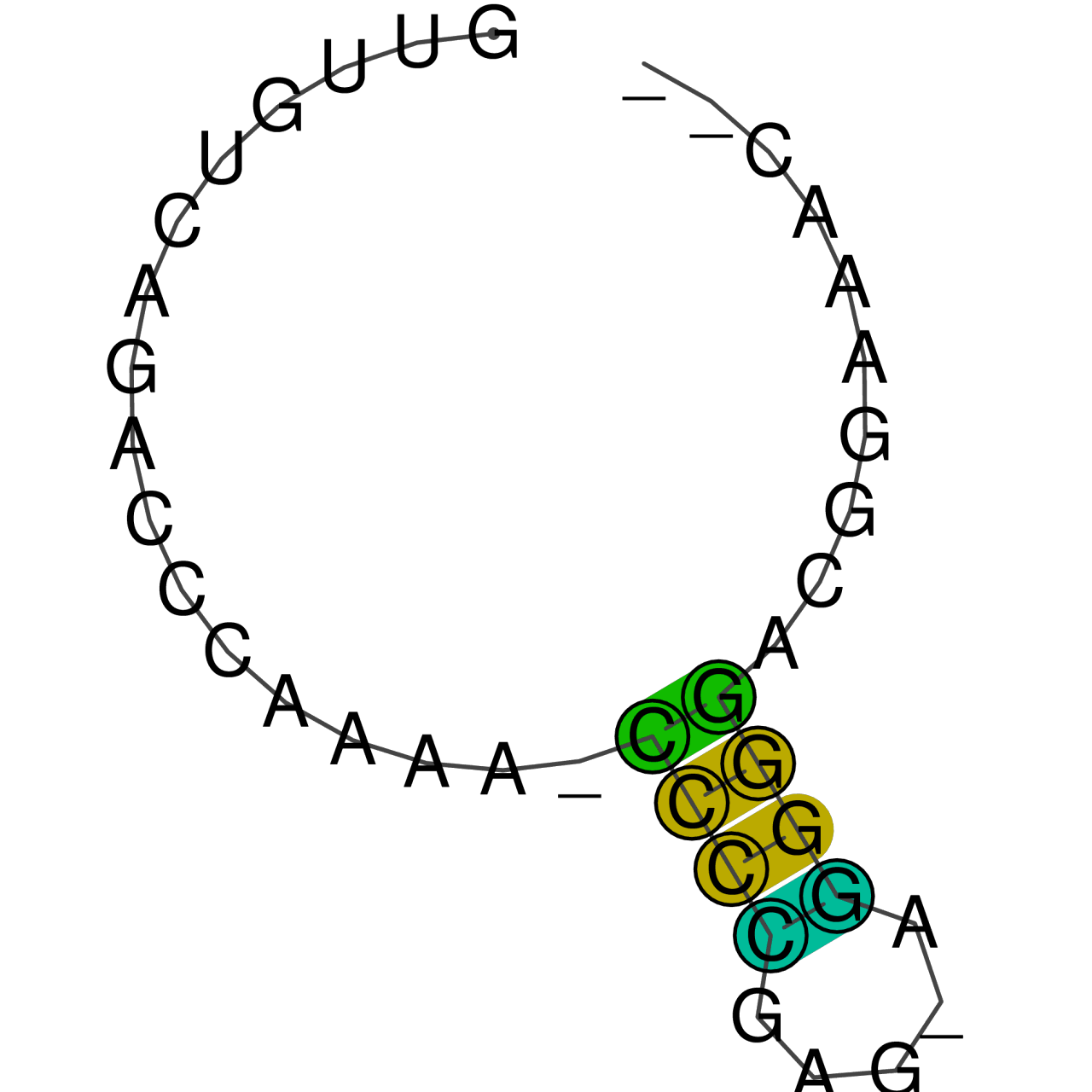 |
| MTUB_CR2 | *Mycobacterium* *tuberculosis* H37Rv | GTCGTCAGACCCAAAACCCCGAGAGGGGACGGAAAC | motif 5 | family 12 | E | 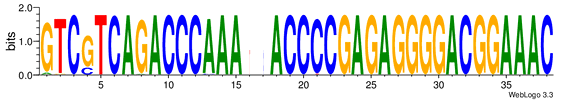 | 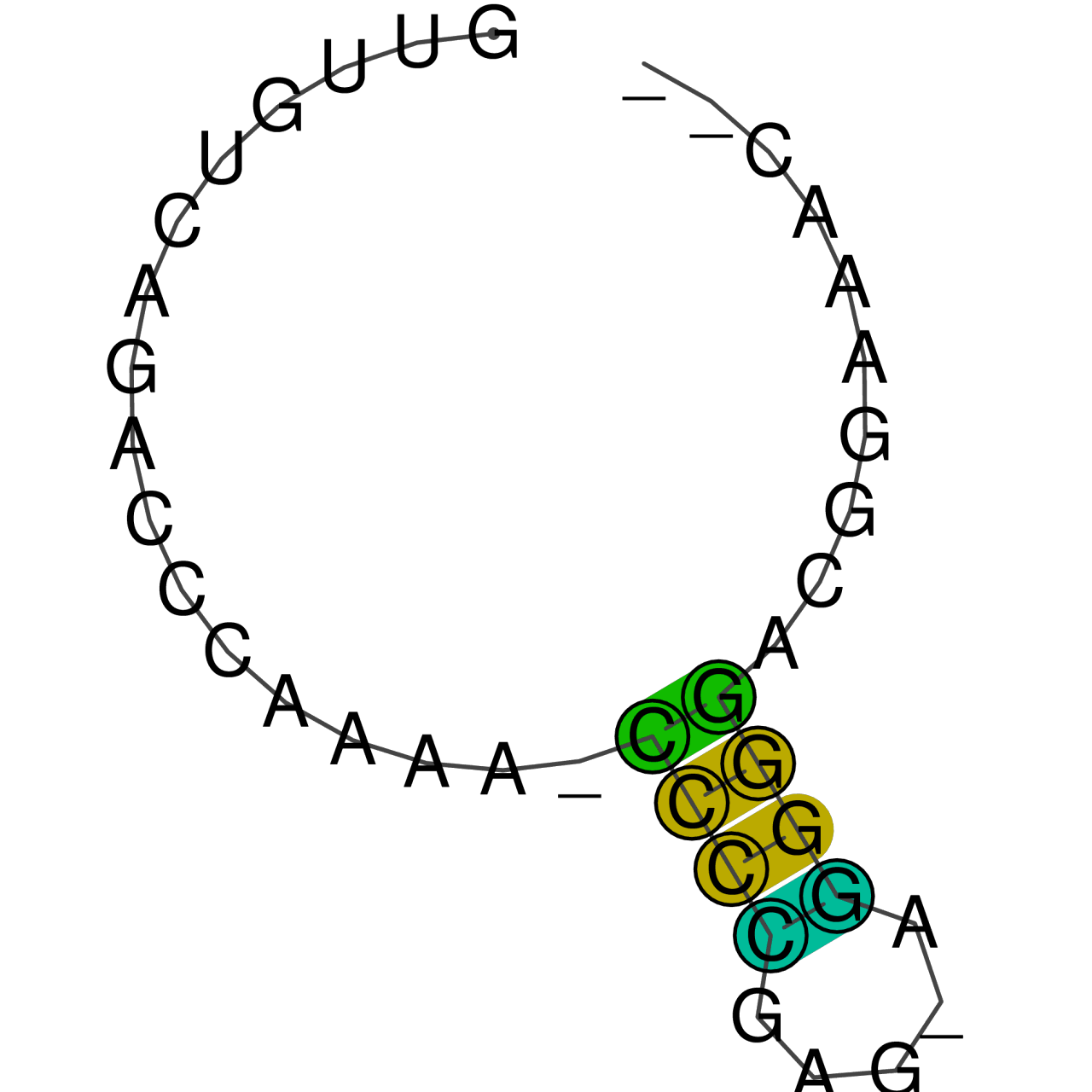 |
| MXEN_CR1 | *Mycobacterium* *xenopi* RIVM700367 | GCCGCAATGGAGCCCAGGCGTTTTCGCCTGGGAGAT | motif 23 | - | ESSSS | - | 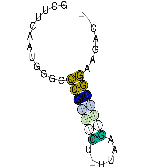 |
| MXEN_CR2 | *Mycobacterium* *xenopi* RIVM700367 | GTGCTCCCCGCGAATGCGGGGATGATCC | - | family 4 | C | 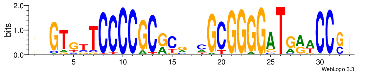 | - |
| TKOR_CR1 | *Mycolicibacillus* *koreensis* strain KCTC 19819 | GGAACACCCCCGCTTGCGCGGGGAGGAG | - | family 2 | B | 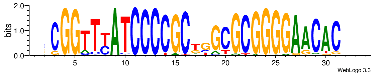 | - |
| TKOR_CR2 | *Mycolicibacillus* *koreensis* strain KCTC 19819 | GGAACACCCCCGCTTGCGCGGGGAGGAG | - | family 2 | B | 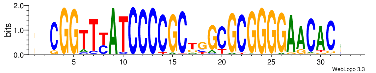 | - |
| TKOR_CR2 | *Mycolicibacillus* *koreensis* strain KCTC 19819 | CTCCTCCCCGCGCAAGCGGGGGTGTTCC | - | family 33 | C | 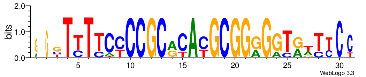 | - |
| TKOR_CR4 | *Mycolicibacillus* *koreensis* strain KCTC 19819 | CTCCTCCCCGCGTAAGCGGGGGTGTTCC | - | family 4 | C | 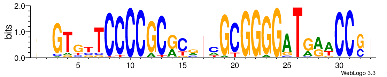 | - |
