# Supplementary figures and images for: Comparative Genomic Analysis of Mycobacteriaceae Reveals Horizontal Gene Transfer-Mediated Evolution of the CRISPR-Cas System in the Mycobacterium tuberculosis Complex
Source: mSystems. 2021 Jan 19;6(1):e00934-20. doi: 10.1128/mSystems.00934-20 (PMC7820667; doi:10.1128/mSystems.00934-20)

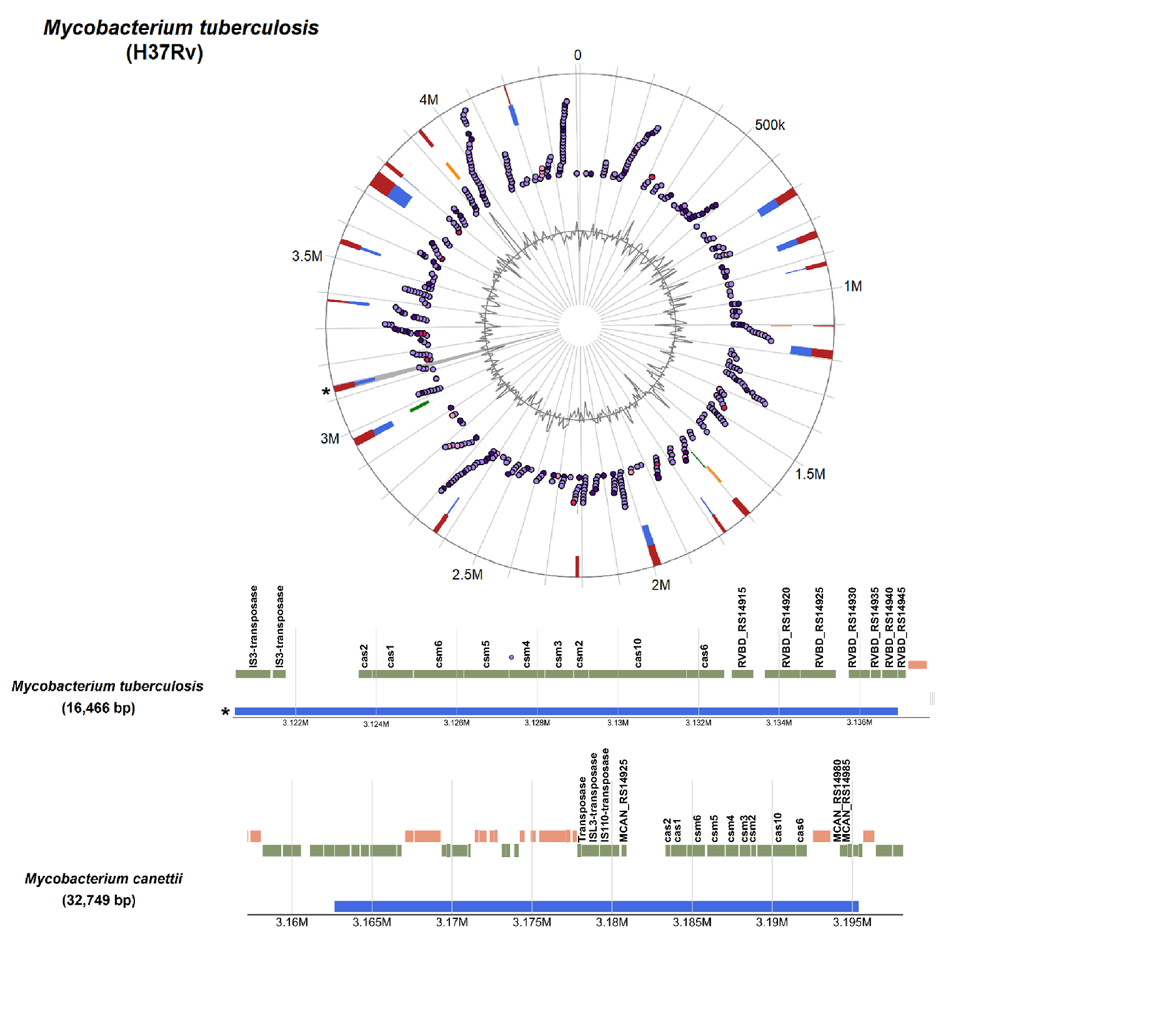

Supplement: FIG S1 [file mSystems.00934-20-sf001.tif]

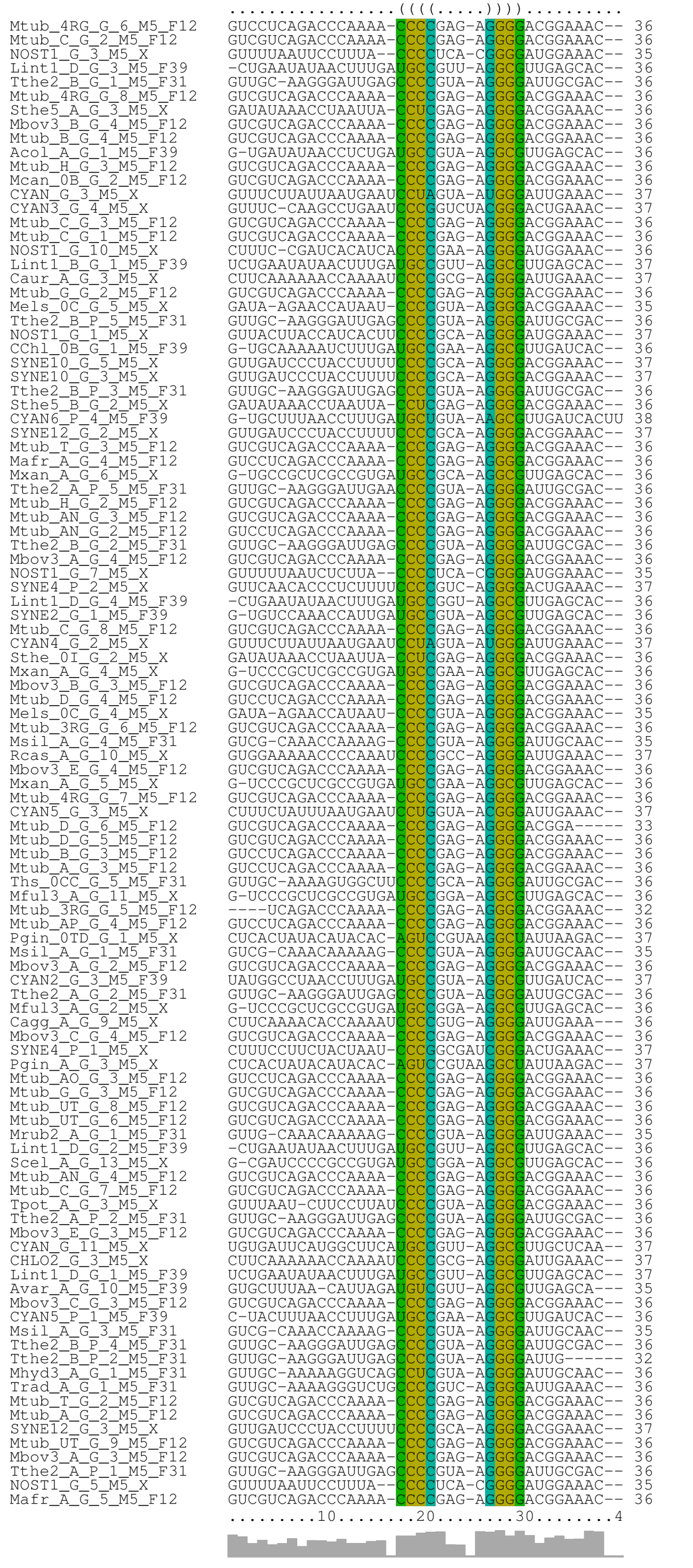

Supplement: FIG S2 [file mSystems.00934-20-sf002.tif]

## a) Cas10 cyclase domain

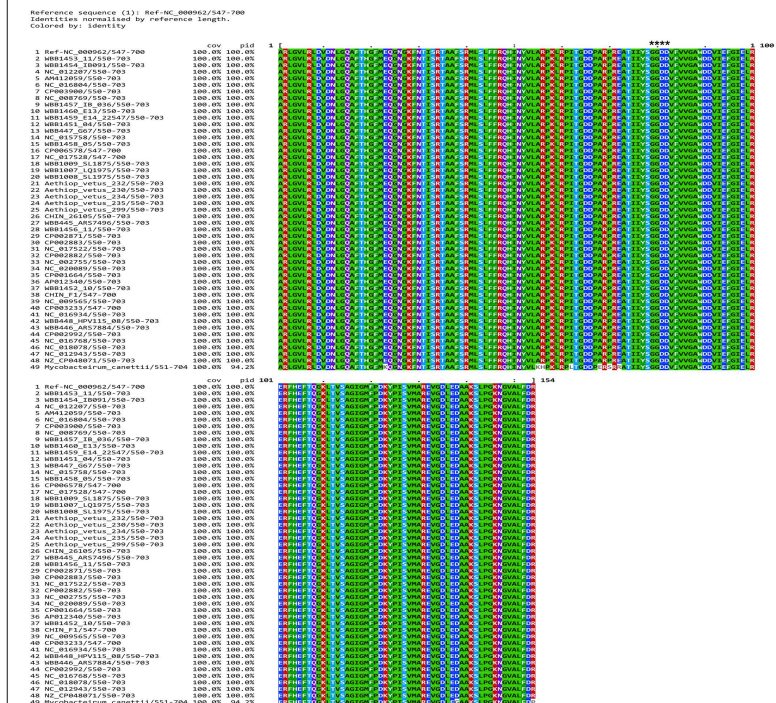

## b) Cas10 HD nuclease domain

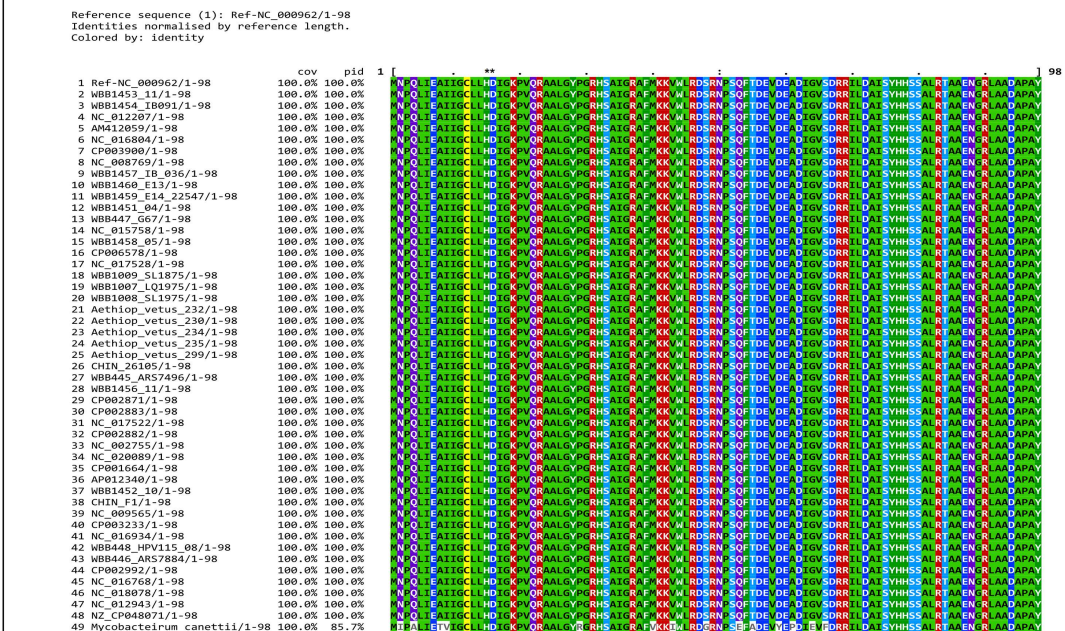

Supplement: FIG S3 [file mSystems.00934-20-sf003.pdf]

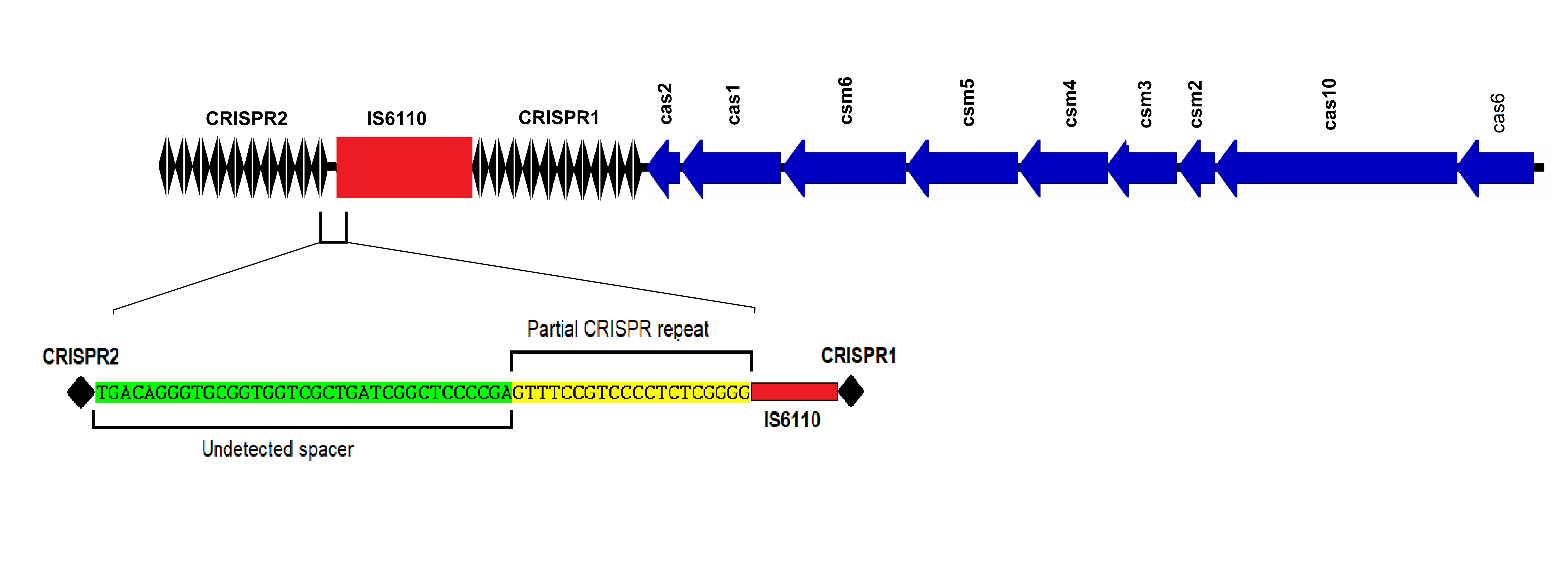

Supplement: FIG S4 [file mSystems.00934-20-sf004.tif]

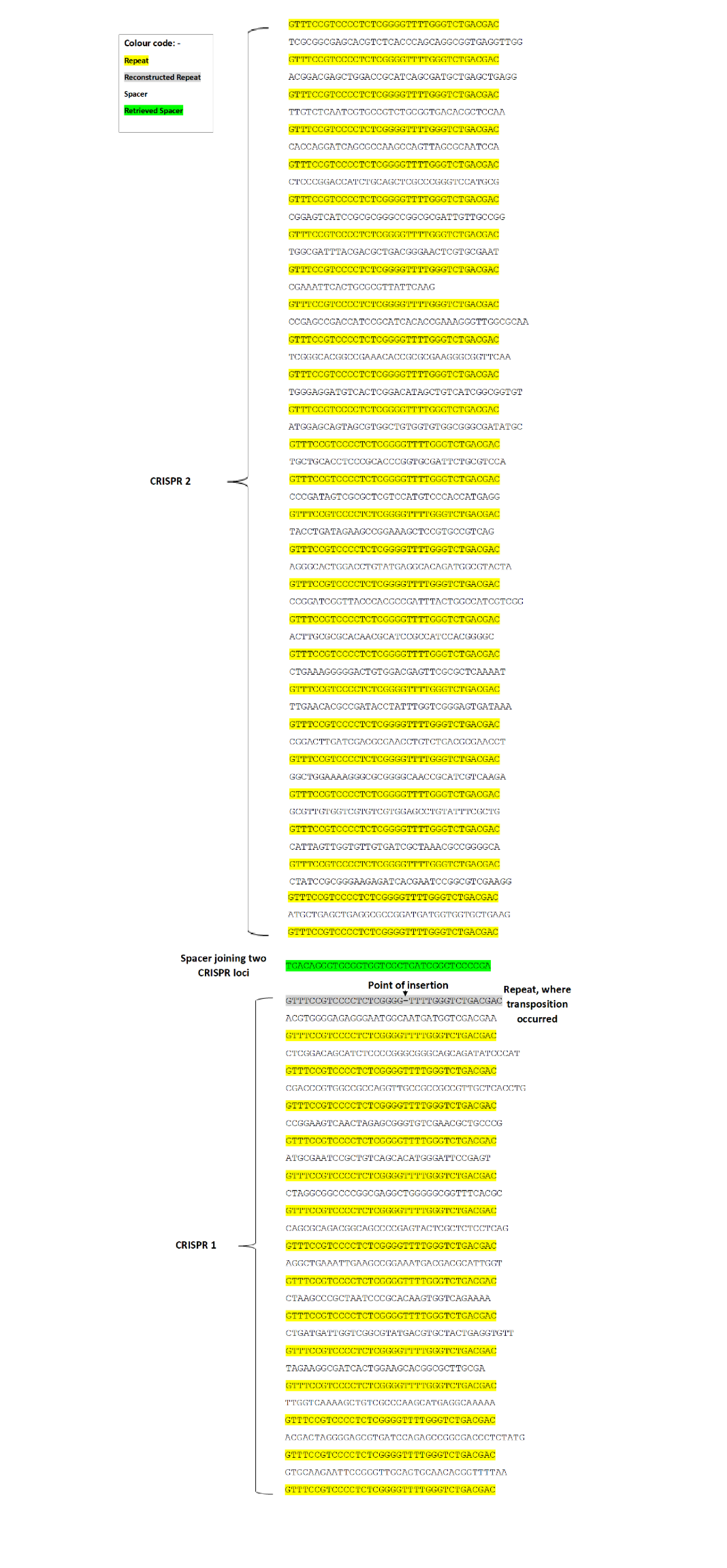

Supplement: FIG S5 [file mSystems.00934-20-sf005.tif]
